# Macrophages programmed by apoptotic cells inhibit epithelial-mesenchymal transition in lung alveolar epithelial cells via PGE_2_, PGD_2_, and HGF

**DOI:** 10.1038/srep20992

**Published:** 2016-02-15

**Authors:** Young-So Yoon, Ye-Ji Lee, Youn-Hee Choi, Young Mi Park, Jihee Lee Kang

**Affiliations:** 1 Department of Physiology, School of Medicine, Ewha Womans University, Seoul 158-710, Korea; 2 Tissue Injury Defense Research Center, School of Medicine, Ewha Womans University, Seoul 158-710, Korea; 3 Department of Molecular medicine, School of Medicine, Ewha Womans University, Seoul 158-710, Korea

## Abstract

Apoptotic cell clearance results in the release of growth factors and the action of signaling molecules involved in tissue homeostasis maintenance. Here, we investigated whether and how macrophages programmed by apoptotic cells inhibit the TGF-β1-induced Epithelial-mesenchymal transition (EMT) process in lung alveolar epithelial cells. Treatment with conditioned medium derived from macrophages exposed to apoptotic cells, but not viable or necrotic cells, inhibited TGF-β1-induced EMT, including loss of E-cadherin, synthesis of N-cadherin and α-smooth muscle actin, and induction of EMT-activating transcription factors, such as Snail1/2, Zeb1/2, and Twist1. Exposure of macrophages to cyclooxygenase (COX-2) inhibitors (NS-398 and COX-2 siRNA) or RhoA/Rho kinase inhibitors (Y-27632 and RhoA siRNA) and LA-4 cells to antagonists of prostaglandin E_2_ (PGE_2_) receptor (EP4 [AH-23848]), PGD_2_ receptors (DP1 [BW-A868C] and DP2 [BAY-u3405]), or the hepatocyte growth factor (HGF) receptor c-Met (PHA-665752), reversed EMT inhibition by the conditioned medium. Additionally, we found that apoptotic cell instillation inhibited bleomycin-mediated EMT in primary mouse alveolar type II epithelial cells *in vivo*. Our data suggest a new model for epithelial cell homeostasis, by which the anti-EMT programming of macrophages by apoptotic cells may control the progressive fibrotic reaction via the production of potent paracrine EMT inhibitors.

Idiopathic pulmonary fibrosis (IPF) is a progressive and generally fatal disorder of unknown etiology^1^. IPF is an irreversible process characterized by alveolar epithelial cell injury, fibroblast accumulation, and differentiation to myofibroblasts^2^. One of the histopathological hallmarks of IPF are fibroblast foci, which consist of activated fibroblast aggregates that produce excess extracellular matrix (ECM) proteins, such as collagens I and III, fibronectin, and lamin, within the alveolar space at the site of epithelial cell loss^3^,^4^,^5^. Originally, local tissue myofibroblasts were cited as the primary source of ECM components following injury^6^; however, it is now thought that fibroblasts can be derived from multiple sources^7^. In addition to resident mesenchymal cells, myofibroblasts are derived from differentiation of bone marrow progenitor cells and the epithelial-mesenchymal transition (EMT) process^7^,^8^,^9^,^10^. Emerging evidence suggests that the EMT process is a major event in IPF pathogenesis^11^,^12^. Although several drugs are currently used to treat the symptoms and slow progression of IPF to little effect, no proven, efficacious treatment currently exists^13^.

Apoptotic cell clearance by tissue macrophages and non-professional phagocytes is an essential process in tissue homeostasis, immunity, and inflammation resolution. Surface changes on the apoptotic cells trigger the recognition by phagocytes^14^,^15^,^16^,^17^,^18^,^19^,^20^. Widely distributed surface ligand on apoptotic cells is phospholipid phosphatidylserine or calreticulin^15^,^16^, that allow recognition by multiple efferocytic receptors^17^. Some phagocytic receptors interact directly with apoptotic cells; however, in many instances, apoptotic cells interact indirectly with apoptotic cells through bridging molecules, including milk fat globule EGF-8 (MFGE8), protein S and Gas6^18^. Engagement of efferocytic receptors initiates signaling events modulated by two main complexes, CrkII/ELMO/Dock180^19^ or ABCA1/GULP^20^, both resulting in activation of Rac1, which facilitates cytoskeletal rearrangement for engulfment^18^. The regulation of efferocytosis is tightly controlled by Rho-family GTPases; thus, Rac-1 promotes and RhoA inhibits this process^21^,^22^,^23^,^24^.

Apoptotic cell recognition by macrophages actively leads to production of anti-inflammatory mediators, such as transforming growth factor (TGF)-β, interleukin (IL)-10, and prostaglandin E_2_ (PGE_2_)^16^,^25^. Interactions between apoptotic and phagocytic cells play important roles in the regeneration and repair of damaged tissues by inducing growth maintenance factors, such as vascular endothelial growth factor (VEGF), hepatocyte growth factor (HGF), and PGE_2_, which can reconstitute the damaged tissue leading to decreased fibroproliferative sequelae^14^,^26^,^27^,^28^,^29^. We demonstrated that *in vivo* apoptotic cell exposure resulted in enhanced HGF and cyclooxygenase (COX)-2 expression and PGE_2_ secretion until the late fibrotic phase in bleomycin-induced lung injury^30^,^31^. We also showed that interaction with apoptotic cells induces persistent COX-2/PGE_2_ and HGF upregulation in a positive feedback loop, which propagates anti-inflammatory, anti-apoptotic, and anti-fibrotic signaling. Importantly, many studies provide evidence that the HGF-associated COX-2/PGE_2_ pathway is a potent inhibitor of EMT with fibrotic remodeling^32^,^33^,^34^,^35^. However, the impact of the COX-2 and HGF pathways on the prevention of EMT progression in the context of enhanced apoptotic cell recognition and clearance has not been studied.

In the present study, we used *in vitro* co-incubation assays to demonstrate that macrophages programmed by apoptotic cells modulate EMT in lung epithelial cells. We also determined how COX-2-derived PGE_2_ and PGD_2_, as well as RhoA-dependent HGF secretion from macrophages in response to apoptotic cells, contribute to EMT inhibition. Moreover, we provided *in vivo* evidence that apoptotic cell instillation after bleomycin treatment inhibits EMT in primary mouse alveolar type II epithelial (AT II) cells, suggesting a potential therapeutic option for IPF treatment.

## Results

### Macrophages exposed to apoptotic cells counteract TGF-β-induced EMT in lung and kidney epithelial cells

**Figure 1:**
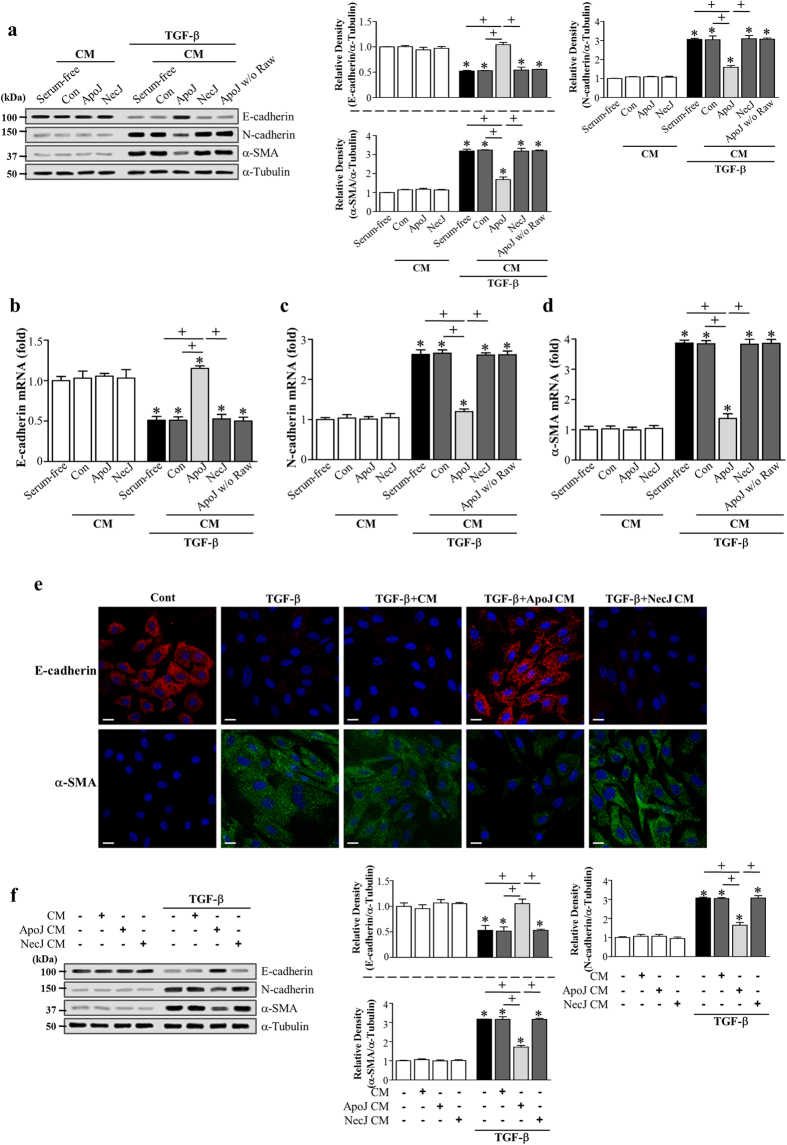
Conditioned medium from RAW 264.7 cells exposed to apoptotic cells reduced TGF-β1-induced EMT in lung epithelial cells. RAW 264.7 cells were stimulated with apoptotic (ApoJ) or necrotic (NecJ) Jurkat cells for 20 h. Conditioned medium (CM) was added to LA-4 cells (**a–e**) or primary mouse alveolar type II epithelial (AT II) cells (**f**) in the absence or presence of 10 ng/ml TGF-β1 for 72 h. (**a**,**f**) Immunoblots of total cell lysates were performed with anti-E-cadherin, N-cadherin, or α-SMA antibodies. Right: Densitometric analysis of the indicated EMT markers’ relative abundances. (**b–d**) The amount of EMT markers’ mRNA in LA-4 cell samples was analyzed by real-time PCR and normalized to that of *Hprt* mRNA. Values represent the mean ± s.e.m. of three independent experiments. *P < 0.05; compared with control or conditioned medium from RAW cells with ApoJ, or NecJ cells at 72 h after TGF-β1 treatment; ^+^*P* < 0.05 as indicated. (**e**) Immunofluorescence staining for E-cadherin (*red*) or α-SMA (*green*) in LA-4 cells. Scale bars = 20 μm. Results are representative of three independent experiments.

TGF-β1 activation is a critical signaling element in EMT and plays a central role in pulmonary fibrosis pathogenesis. Thus, we assessed the impact of *in vitro* phagocyte exposure to apoptotic cells on TGF-β1-induced EMT in murine AT II-like lung epithelial (LA-4) cells. TGF-β1 exposure for 2–3 days caused LA-4 cells to undergo EMT, during which cells acquired a spindle-like shape (Supplementary Fig. S1a). Additionally, adherens junction protein E-cadherin expression was decreased, whereas the expression of N-cadherin and α-smooth muscle actin (SMA), a marker of myofibroblast differentiation, was upregulated (Supplementary Fig. S1b-d). Treatment with conditioned medium derived from a murine macrophage cell line (RAW 264.7) exposed to apoptotic Jurkat cells for 20 h (ApoJ-exposed CM) inhibited TGF-β1-induced EMT in LA-4 cells, based on morphologic cellular alteration (Supplementary Fig. S1a) and EMT marker expression profiles at both the protein (Fig. 1a) and mRNA level (Fig. 1b–d). These EMT marker changes weakened inversely as the conditioned medium was diluted 1:2 and 1:4 with *X-VIVO* medium (Supplementary Fig. S1e). However, this inhibitory effect was not observed with conditioned media derived from co-culture with control, viable (ViaJ-exposed CM; Supplementary Fig. S1e) or necrotic Jurkat cells (NecJ-exposed CM). In addition, culture supernatant from apoptotic Jurkat cells alone did not induce an anti-EMT effect. Immunofluorescence using E-cadherin (red) and α-SMA (green) monoclonal antibodies was performed to validate EMT marker protein changes. Similar to the western data, the TGF-β1-induced decrease in E-cadherin expression and increase in α-SMA expression in LA-4 cells were reversed by ApoJ-exposed CM, but not NecJ-exposed CM (Fig. 1e). We also confirmed the inhibitory effect of the ApoJ-exposed CM on TGF-β1-induced EMT in primary mouse AT II cells (Fig. 1f) as well as HEK-293 human embryonic kidney epithelial cells (Supplementary Fig. S2a).

To demonstrate that the anti-EMT effect of the conditioned medium was not restricted to apoptotic T cells, other apoptotic cell types, such as LA-4 epithelial cells, were exposed to RAW 264.7 cells, and the conditioned medium was added to LA-4 cells in the presence of TGF-β1. That conditioned medium exerted similar inhibitory effects on EMT marker expression in LA-4 cells (Supplementary Fig. S2b). Similar effects were also observed with apoptotic human Jurkat T cells and mouse thymocytes (Supplementary Fig. S2c).

**Figure 2:**
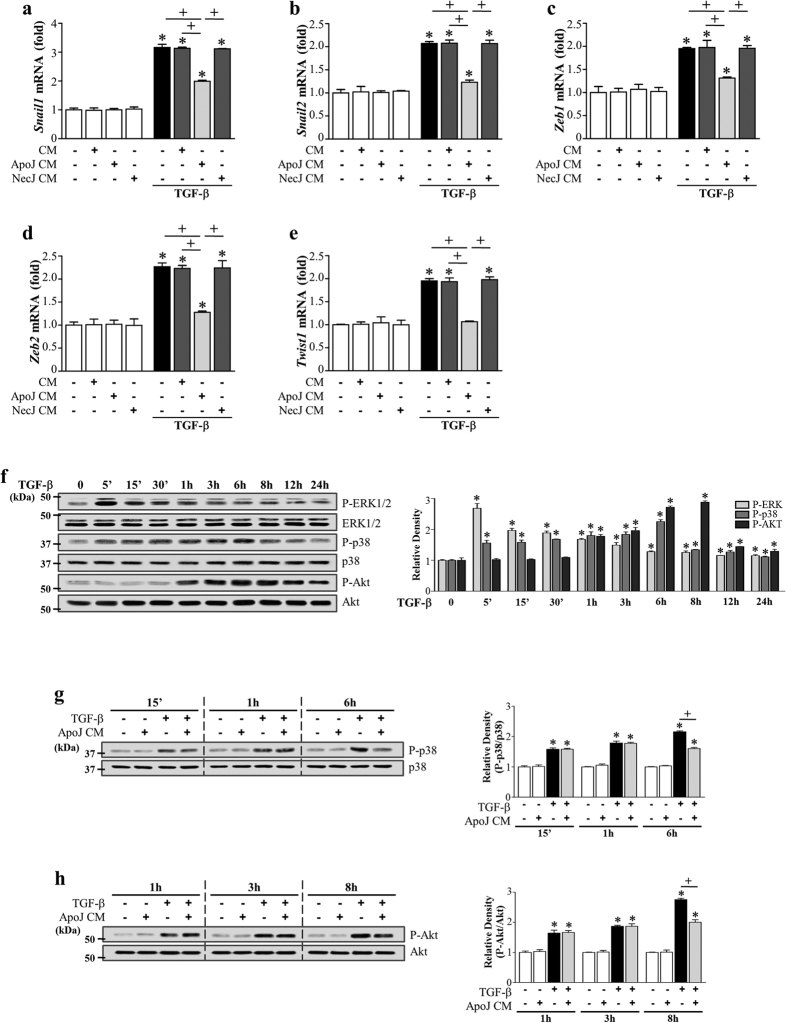
Conditioned medium from RAW 264.7 cells exposed to apoptotic cells reduced TGF-β1-induced EMT-regulating transcription factor expression and blocked Smad-independent TGF-β1 signaling in LA-4 cells. RAW 264.7 cells were stimulated with apoptotic (ApoJ) or necrotic (NecJ) Jurkat cells for 20 h. Conditioned medium (CM) was added to LA-4 cells in the absence or presence of 10 ng/ml TGF-β1 for 72 h or the indicated time. (**a–e**) The amount of *Snail1/2, Zeb1/2, and Twist1* mRNA in LA-4 cell samples was analyzed by real-time PCR and normalized to that of *Hprt* mRNA. (**f**–**h**) Western blot analysis of the relative amounts of total and phosphorylated ERK, p38 MAP kinase, and Akt protein in the indicated samples over time. Densitometric analysis of the relative phosphorylated protein abundances, normalized to that of total protein. Data in all bar graphs are the mean ± s.e.m. of three independent experiments. *P < 0.05 compared with control; ^+^*P* < 0.05 as indicated.

The loss of epithelial markers and acquisition of mesenchymal features is achieved through the well-orchestrated actions of the Snail, ZEB, and Basic helix-loop-helix transcription factor families^36^,^37^. Thus, we examined whether ApoJ-exposed CM inhibited enhanced expression of these transcription factors in LA-4 cells with TGF-β1 treatment. The ApoJ-exposed CM inhibited TGF-β1-induced mRNA expression of *Snail1/2, Zeb1/2*, and *Twist1* in LA-4 cells (Fig. 2a–e), whereas the control, or NecJ-exposed CM did not affect transcription factor induction.

### Macrophages exposed to apoptotic cells antagonize Smad-independent TGF-β1 signaling in LA-4 cells

To further explore the intracellular signal transduction mechanism, we examined the effects of the conditioned medium on the canonical Smad-dependent TGF-β1 signaling pathway. The ApoJ-exposed CM did not affect TGF-β1-mediated phosphorylation of Smad2 and Smad3 in LA-4 cells(Supplementary Fig. S3a,b). Next, we examined Smad-independent TGF-β1 signaling pathways, such as mitogen-activated protein (MAP) kinase and phosphoinositide 3-kinase (PI3K)/protein kinase B (Akt) pathways. Extracellular signal-regulated kinase (ERK) was enhanced at 5 min and then declined slightly, and p38 MAP kinase, and Akt phosphorylation was enhanced up to 6, and 8–12 h after TGF-β1 stimulation, respectively, in LA-4 cells (Fig. 2f), whereas c-Jun N-terminal kinase (JNK) phosphorylation was not enhanced over the 24-h observation period (data not shown). ApoJ-exposed CM treatment partially inhibited TGF-β1-induced phosphorylation of p38 MAP kinase and Akt (Fig. 2g,h); however, it had no effect on ERK phosphorylation (Supplementary Figure S3c). These data suggest that the bioactive mediators secreted from apoptosis-stimulated macrophages partially block Smad-independent TGF-β signaling, including the p38 MAP kinase and Akt pathways, in LA-4 cells.

### Direct exposure of LA-4 epithelial cells to apoptotic cells does not inhibit EMT in LA-4 cells

**Figure 3:**
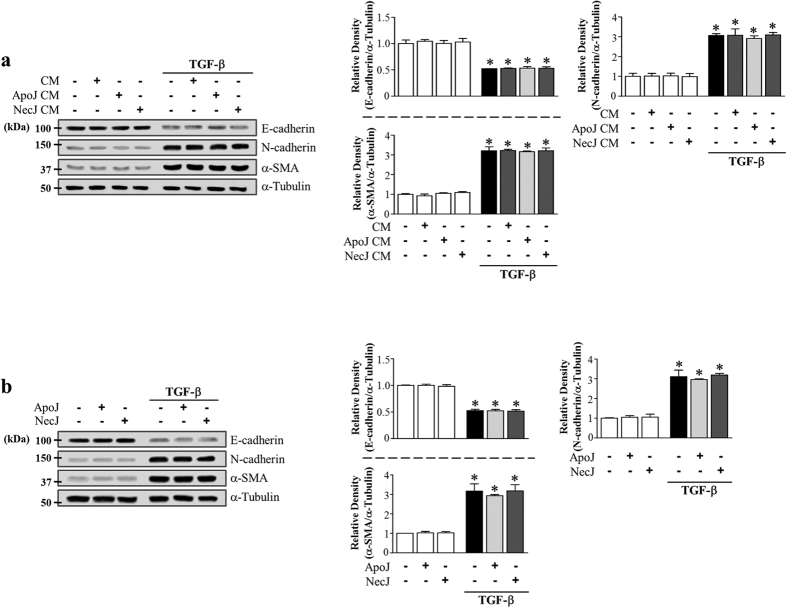
Effect of direct exposure of LA-4 cells to apoptotic cells on TGF-β1-induced EMT. (**a**) LA-4 cells were stimulated with apoptotic (ApoJ) or necrotic (NecJ) Jurkat cells for 20 h. Conditioned medium (CM) was added to LA-4 cells in the presence or absence of 10 ng/ml TGF-β1. (**b**) LA-4 cells were directly exposed to ApoJ or NecJ cells in the presence or absence of 10 ng/ml TGF-β1. (**a**,**b**) After 72 h, western blot analysis of EMT markers in LA-4 cells. Values represent the mean ± s.e.m. of three independent experiments. *P < 0.05 compared with control; ^+^*P* < 0.05 as indicated.

Non-professional phagocytes, such as epithelial cells, are capable of recognizing apoptotic cells, inducing engulfment, and delivering anti-inflammatory, anti-immunogenic, and repair signals to the immediate environment^25^,^38^. Thus, we examined whether ApoJ-exposed CM from LA-4 epithelial cells inhibited TGF-β1-induced EMT. Neither conditioned medium nor direct exposure to apoptotic Jurkat cells inhibited TGF-β1-induced EMT in LA-4 cells (Fig. 3a,b, respectively). These data indicate that TGF-β1-induced EMT inhibition requires bioactive mediators secreted by professional phagocytes, such as macrophages, which are functionally altered by apoptotic cell stimulation.

### COX-2-derived PGE_2_ and PGD_2_ secretion from macrophages induced by apoptotic cells mediates EMT inhibition

**Figure 4:**
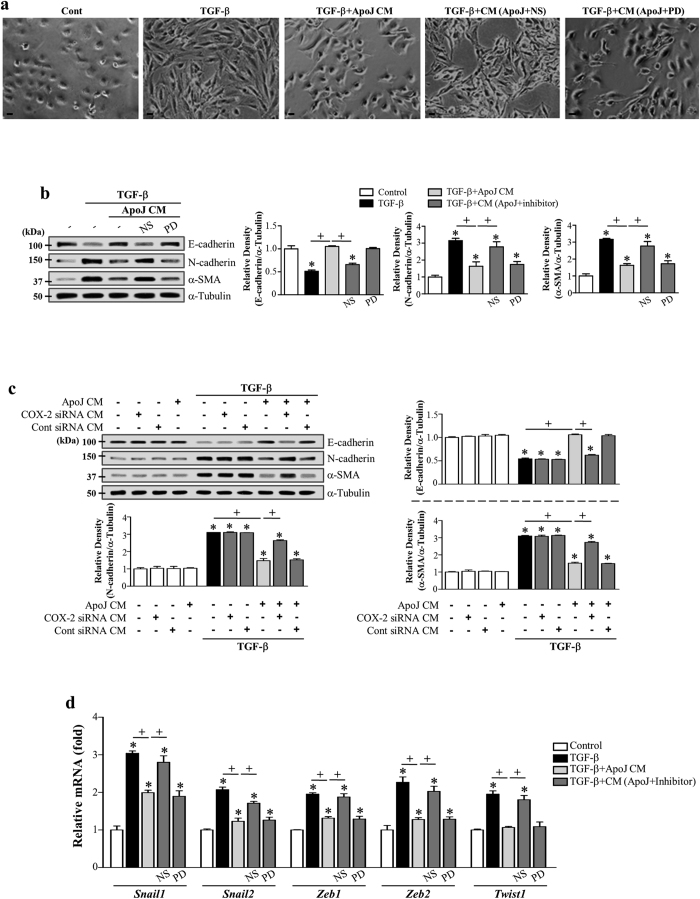
COX-2 signaling in RAW 264.7 cells in response to apoptotic cells mediates EMT inhibition in LA-4 cells. (**a**,**b**,**e**) RAW 264.7 cells were pretreated with 10 μM NS-398 or 10 μM PD-146176 for 1 h and then stimulated with apoptotic Jurkat cells (ApoJ) for 20 h. (**c**) RAW cells were transfected with COX-2 siRNA or control vehicle (siRNA-GFP) for 6 h, then incubated with ApoJ for 20 h. Conditioned medium (CM) was added to LA-4 cells in the presence of TGF-β1 for 72 h. (**a**) Morphological changes in the cells were examined by phase-contrast microscopy. Scale bars = 50 μm. (**b**,**c**) Immunoblots of total cell lysates were performed with anti-E-cadherin, N-cadherin, or α-SMA antibodies. Right: Densitometric analysis of the indicated EMT markers’ relative abundances. (**d**) The amounts of the *Snail1/2, Zeb1/2, and Twist1* mRNAs in LA-4 cell samples were analyzed by real-time PCR and normalized to that of *Hprt* mRNA. Values represent the mean ± s.e.m. of three independent experiments. *P < 0.05 compared with control; ^+^*P* < 0.05 as indicated.

The COX-2/PGE_2_ and PGD_2_ pathways are potent EMT inhibitors in renal epithelial cells^32^,^39^. We showed that PGE_2_ and PGD_2_ production in RAW 264.7 cells is enhanced by exposure to apoptotic cells, predominantly via inducing COX-2 expression (Supplementary Fig. S4a,b)^40^. Thus, we investigated whether COX-2-derived PGE_2_ and PGD_2_, secreted from ApoJ-exposed macrophages, mediate anti-EMT effects in LA-4 cells. First, to determine the functional relevance of COX-2 signaling in macrophage-dependent anti-EMT effects, RAW 264.7 cells were pretreated with the highly selective COX-2 inhibitor NS-398 or COX-2-specific siRNA and incubated with apoptotic Jurkat cells for 20 h. NS-398 pre-treatment reversed the effect of conditioned medium on TGF-β1-induced morphology (Fig. 4a). The RAW 264.7 cells were transfected with COX-2 or COX-1 specific siRNAs or with a negative-control siRNA and then were cultured for 6 or 48 h, respectively. The negative-control siRNA did not alter COX-2 and COX-1 protein amounts in cells (Supplementary Fig. S4c,d). The abundances of COX-2 or COX-1 proteins were specifically decreased by ~100 or 90%, respectively, in cells transfected with COX-2 - or COX-1-specific siRNAs at 20 h after apoptotic cell exposure compared to those in naïve RAW264.7 cells. NS-398 or COX-2 gene knockdown reversed the inhibition of TGF-β1-induced EMT in LA-4 cells, E-cadherin loss, and synthesis of α-SMA and N-cadherin at both the gene (Supplementary Fig. S4e) and protein level (Fig. 4b,c). In contrast, COX-1 expression knockdown using COX-1-specific siRNA transfection did not reverse the inhibition of TGF-β1-induced EMT in LA-4 cells (Supplementary Fig. S4f). Moreover, NS-398 reversed reduction of TGF-β1-induced mRNA expression of *Zeb1/2, Snail1/2*, and *Twist1* in LA-4 cells by the ApoJ-exposed CM (Fig. 4d). The 15-lipoxygenase inhibitor PD-146176 did not reverse TGF-β-induced EMT inhibition by the conditioned medium in LA-4 cells (Supplementary Figure S4e; Fig. 4b,d). Quantitative morphometric analysis showed that NS-398, but not PD-146176, reversed the reduction of degree of elongated cell morphology or morphological index in LA4-cells exposed to ApoJ-exposed CM (Supplementary Fig. S5a).

**Figure 5:**
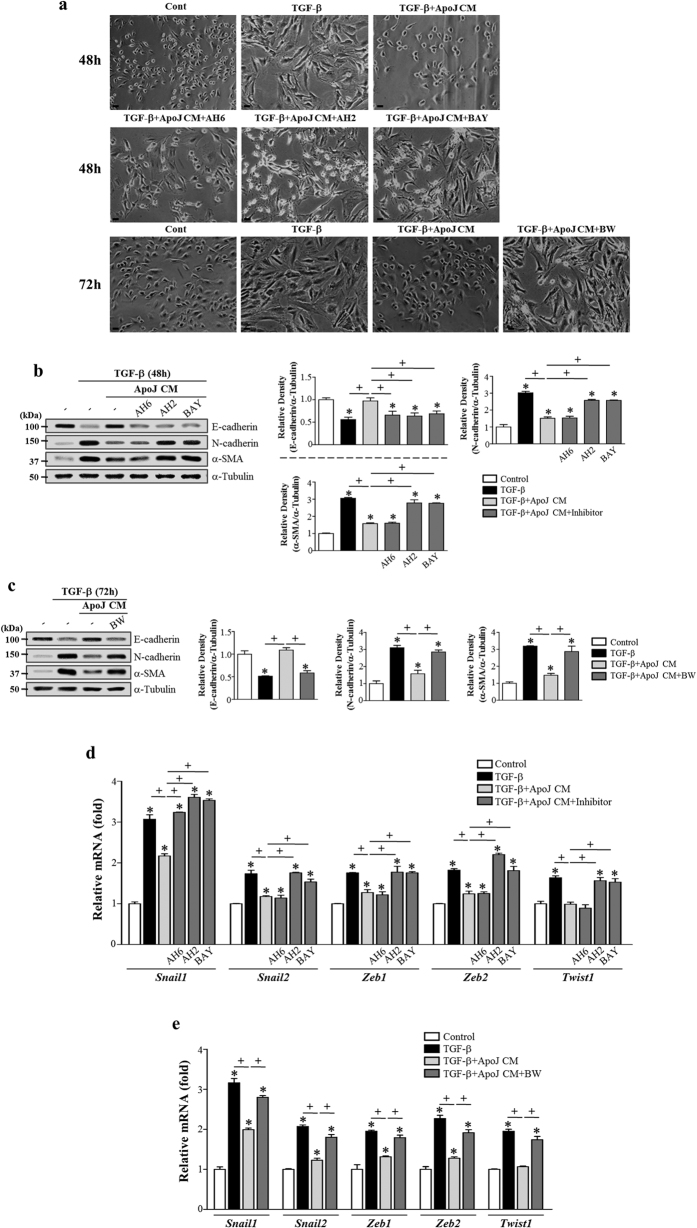
PGE_2_ and PGD_2_ from RAW 264.7 cells in response to apoptotic cells mediate EMT inhibition in LA-4 cells via their receptors. RAW 264.7 cells were stimulated with apoptotic Jurkat cells (ApoJ) for 20 h. Conditioned medium (CM) was added to LA-4 cells in the presence of TGF-β1 with or without antagonists of EP2 (AH-6809), EP4 (AH-23848), DP1 (BW-A868C), or DP2 (BAY-u3405) at 10 μM. After 48 or 72 h, morphological changes in the cells were examined by phase-contrast microscopy (Scale bars = 50 μm) (**a**), and immunoblots of total cell lysates were performed with anti-E-cadherin, N-cadherin, or α-SMA antibodies. Right: Densitometric analysis of the indicated EMT markers’ relative abundances (**b**,**c**). (**d**,**e**) The amounts of *Snail1/2, Zeb1/2, and Twist1* mRNAs in LA-4 cell samples were analyzed by real-time PCR and normalized to that of *Hprt* mRNA. Values represent the mean ± s.e.m. of three independent experiments. *P < 0.05 compared with control; ^+^*P* < 0.05 as indicated.

To determine whether macrophage secretion of COX-2-derived PGE_2_ and PGD_2_ mediates anti-EMT effects, ApoJ-exposed CM was added to TGF-β1-stimulated LA-4 cells in the presence of PGE_2_- or PGD_2_-specific receptor antagonists, such as antagonists of E-prostanoid-2 receptor (EP2) (AH-6809), EP4 (AH-23848), DP1 (BW-A868C), or DP2 (BAY-u3405). The EP2 antagonist AH-6809 affected weakly TGFβ1-induced morphologic change (Fig. 5a), but only showed an inhibitory effect with respect to inhibiting the loss of E-cadherin mRNA and protein (Supplementary Fig. S5b; Fig. 5b) and *Snail1* mRNA expression (Fig. 5d). Other antagonists, such as AH-23848, BW-A868C, and BAY-u3405, significantly reversed anti-EMT effects, including reversing spindle-like morphology (Fig. 5a) and inhibiting E-cadherin loss, reducing synthesis of N-cadherin and α-SMA at the gene (Supplementary Fig. S5b,c) and protein (Fig. 5b,c) level, and restoring the mRNA abundance of *Snail 1/2, Zeb1/2,* and *Twist1* in LA-4 cells (Fig. 5d,e). Quantitative morphometric analysis also showed that AH-23848, BW-A868C, and BAY-u3405 but not AH-6809 reversed the reduction of morphological index in LA4-cells exposed to ApoJ-exposed CM (Supplementary Fig. S5d,e). These data indicate that anti-EMT effects were mediated by COX-2-derived PGE_2_ and PGD_2_ signaling, primarily through the EP4, DP1, and DP2 receptors in LA-4 cells.

### RhoA-dependent HGF secretion from macrophages programed by apoptotic cells mediates EMT inhibition

**Figure 6:**
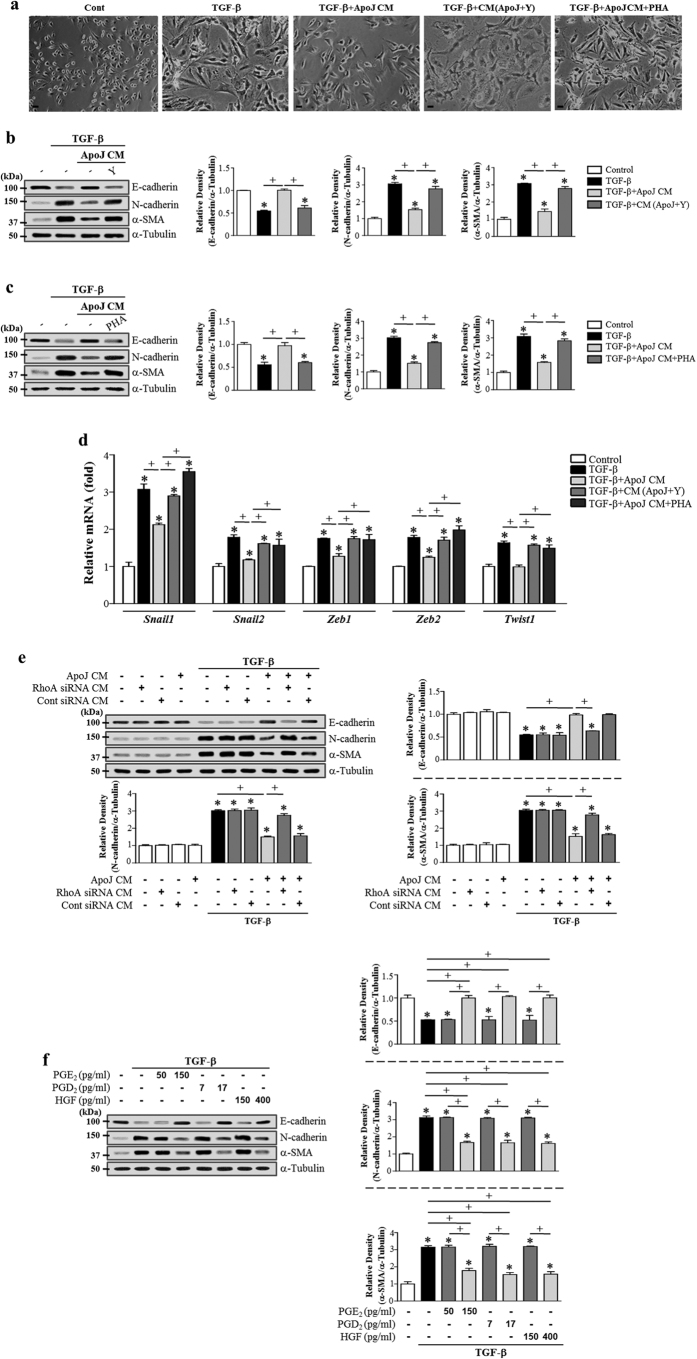
RhoA-dependent HGF secretion from RAW 264.7 cells and exogenous PGE_2_, PGD_2_, and HGF mediate EMT reduction in LA-4 cells. (**a**,**b**,**d**) RAW 264.7 cells were pretreated with 30 μM Y-27632 for 1 h and then stimulated with apoptotic Jurkat cells (ApoJ) for 20 h. (**e**) RAW cells were transfected with RhoA siRNA or control vehicle (siRNA-GFP) for 24 h, then incubated with ApoJ for 20 h. Conditioned medium (CM) was added to LA-4 cells in the presence of TGF-β1 for 48 h. (**a,c,d**) RAW 264.7 cells were stimulated with ApoJ for 20 h. CM was added to LA-4 cells in the presence of TGF-β1 with or without the antagonist of c-Met (250 nM PHA-665752). (**f**) PGE_2_ (50 and 150 pg/ml), PGD_2_ (7 and 17 pg/ml), or HGF (150 and 400 pg/ml) was added to LA-4 cell culture in the presence of TGF-β1 for 48 h. (**a**) Morphological changes in the cells were examined by phase-contrast microscopy (Scale bars = 50 μm). (**b**,**c**,**e**,**f**) Immunoblots of total cell lysates were performed with anti-E-cadherin, N-cadherin, or α-SMA antibodies. Right: Densitometric analysis of the indicated EMT markers’ relative abundances. (**d**) The amounts of *Snail1/2, Zeb1/2, and Twist1* mRNAs in LA-4 cell samples were analyzed by real-time PCR and normalized to that of *Hprt* mRNA. Values represent the mean ± s.e.m. of three independent experiments. *P < 0.05 compared with control; ^+^*P* < 0.05 as indicated.

Fibrotic remodeling-associated EMT in alveolar epithelial cells is negatively modulated by HGF^34^. We previously demonstrated that *in vitro* exposure of macrophages to apoptotic cells induced HGF mRNA and protein production via the RhoA-dependent pathway^40^,^41^. Thus, we examined the role of RhoA-dependent HGF secretion from RAW 264.7 cells exposed to apoptotic cells in anti-EMT effects. Inhibition of RhoA/Rho kinase signaling using the Rho kinase inhibitor Y-27632 in macrophages or inhibition of c-Met signaling using PHA-665752 in LA-4 cells reversed TGF-β1-induced EMT inhibition by ApoJ-exposed CM, including reversing spindle-like cellular morphology (Fig. 6a) and reducing E-cadherin loss, synthesis of α-SMA and N-cadherin at the gene (Supplementary Fig. S6a) and protein (Fig. 6b,c) level, and *Zeb1/2, Snail1/2*, and *Twist1* mRNA expression downregulation in LA-4 cells (Fig. 6d). The conditioned medium from RAW 264.7 cells transfected with RhoA siRNA and exposed to apoptotic Jurkat cells also reversed TGF-β1-induced EMT reduction in LA-4 cells (Fig. 6e). In addition, quantitative morphometric analysis showed that the reduced degree of elongated cell morphology in LA4-cells exposed to ApoJ-exposed CM was reversed by Y-27632 and PHA-665752 (Supplementary Fig. S6b). These data strongly suggest that RhoA-dependent HGF production from macrophages in response to apoptotic cells also partially mediates the anti-EMT effect of the conditioned medium via c-Met signaling in LA-4 cells.

### Exogenous PGE_2_, PGD_2_, and HGF inhibit TGF-β1-induced EMT

To confirm that PGE_2_, PGD_2_, and HGF acted in a paracrine manner to induce the anti-EMT effects, we investigated the effect of these soluble mediators at basal (50, 7, and 150 pg/ml, respectively) and stimulation concentrations (150, 17, and 400 pg/ml, respectively)^40^ on LA-4 cells. The conditioned medium derived from macrophages without apoptotic cells contains basal levels of PGE_2_, PGD_2_ and HGF. As expected, each of these soluble mediators at basal concentration did not inhibit TGF-β1-induced changes in EMT markers at both the gene (Supplementary Fig. S7a–c) and protein level (Fig. 6f). Similarly, basal concentrations of combined all these mediators had no inhibitory effects (Supplementary Fig. S7a–d). However, stimulation concentrations of these bioactive molecules inhibited TGF-β1-induced changes in EMT markers at both the gene (Supplementary Fig. S7a–c) and protein level (Fig. 6f) compared with their basal concentrations. These data support our hypothesis that EMT inhibition in LA-4 cells was markedly evoked by apoptotic cell-induced release of PGE_2_, PGD_2_, and HGF. Notably, addition of all these mediators together at the stimulation concentrations to LA-4 cells did not result in synergistic effects (Supplementary Fig. S7a–d). These findings suggest that they may have the same target molecules to inhibit TGF-β1-induced signaling pathway.

To strengthen our conclusion that macrophage-mediated EMT inhibition is exerted partially through the blockage of Smad-independent TGF-β1 signaling, we examined the effect of these soluble molecules on Smad-dependent of independent TGF-β1 signaling in LA-4 cells. As shown in Supplementary Fig. S8), exogenous PGE_2_, PGD_2_, and HGF did not affect Smad-dependent TGF-β1 signaling, but blocked TGF-β1-induced phosphorylation of p38 MAP kinase and Akt in LA-4 cells. These data support our hypothesis that PGE_2_, PGD_2_, and HGF secreted by macrophages in response to apoptotic cells inhibit partially the EMT process through blockage of Smad-independent TGF-β signaling, including the p38 MAP kinase and Akt pathways.

### PGE_2_, PGD_2_, and HGF secretion from murine bone marrow-derived macrophages (BMDM) mediates the anti-EMT effects of the conditioned medium

**Figure 7:**
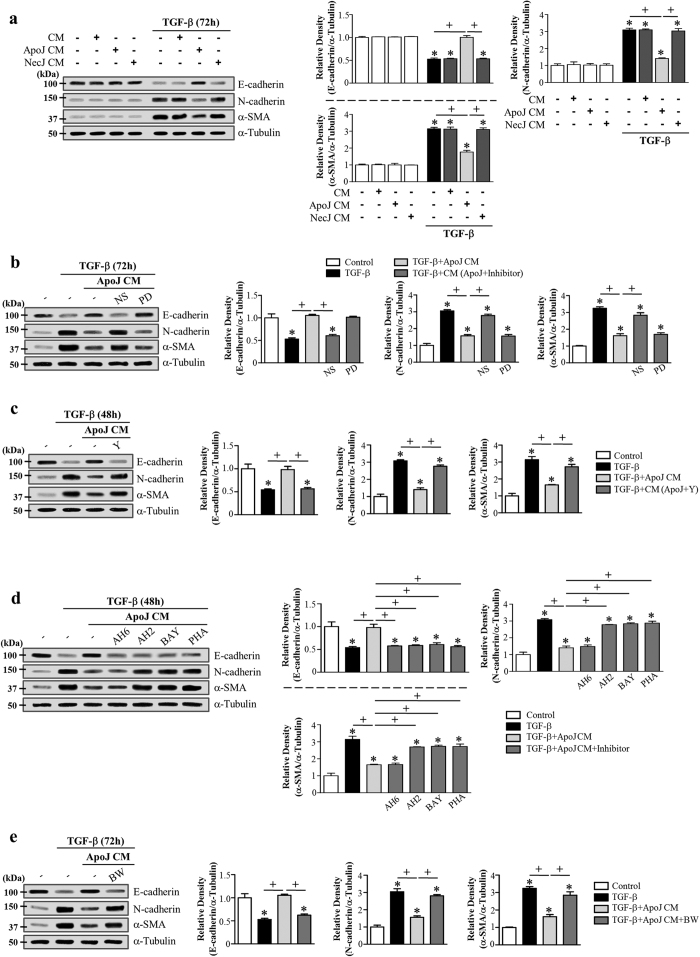
PGE_2_, PGD2, and HGF secretion from murine BMDM mediates EMT reduction in LA-4 cells via their specific receptors. Murine BMDM were stimulated with apoptotic (ApoJ), or necrotic (NecJ) Jurkat T cells for 20 h in the absence (**a**,**d**,**e**) or presence of 10 μM NS-398, 30 μM PD-146176 (**b**), or 30 μM Y-27632 (**c**). Conditioned medium (CM) was added to LA-4 cells in the presence of TGF-β1 for 48 (**c**) or 72 h (**a**,**b**). CM was added to LA-4 cells in the presence of TGF-β1, with or without antagonists of EP2 (10 μM AH-6809), EP4 (10 μM AH-23848), DP1 (10 μM BW-A868C), DP2 (10 μM BAY-u3405), or c-Met (250 nM PHA-665752) for 48 (**d**) or 72 h (**e**). Immunoblots of total cell lysates were performed with anti-E-cadherin, N-cadherin, or α-SMA antibodies. Right: Densitometric analysis of the indicated EMT markers’ relative abundances. Values represent the mean ± s.e.m. of three independent experiments. *P < 0.05 compared with control; ^+^*P* < 0.05 as indicated.

In addition to RAW 264.7 macrophages, we also examined the interaction with primary isolated murine BMDM cultured in the presence of granulocyte macrophage colony-stimulating factor (GM-CSF) and apoptotic or necrotic cells for 20 h. The conditioned medium derived from ApoJ-exposed BMDM substantially inhibited TGF-β1-induced changes in EMT markers in LA-4 cells (Fig. 7a). This inhibitory effect was not observed with conditioned media derived from co-culture with control or necrotic Jurkat cells.

Similarly, treatment of BMDM with COX-2 or Rho kinase inhibitors or LA-4 cells with EP4, DP1, DP2, or c-Met antagonists reversed the conditioned medium-induced reduction of TGF-β1-induced EMT markers at both the gene (Supplementary Fig. S9a–d) and protein level in LA-4 cells (Fig. 7b–e). The EP2 antagonist only reversed the E-cadherin mRNA and protein level reduction (Supplementary Fig. S9c; Fig. 7d). However, 15-lipoxygenase inhibitor PD146176 treatment of BMDM did not reverse the conditioned medium-induced reduction of TGF-β1-induced EMT markers in LA-4 cells (Supplementary Fig. S9a; Fig. 7b). These data indicate that multiple bioactive mediators, such as COX-2/PGE_2_ and /PGD_2_ and RhoA signal-dependent HGF, block the EMT process in LA-4 cells via their specific receptors.

### *In vivo* exposure to apoptotic cells prevents the EMT phenotype

**Figure 8:**
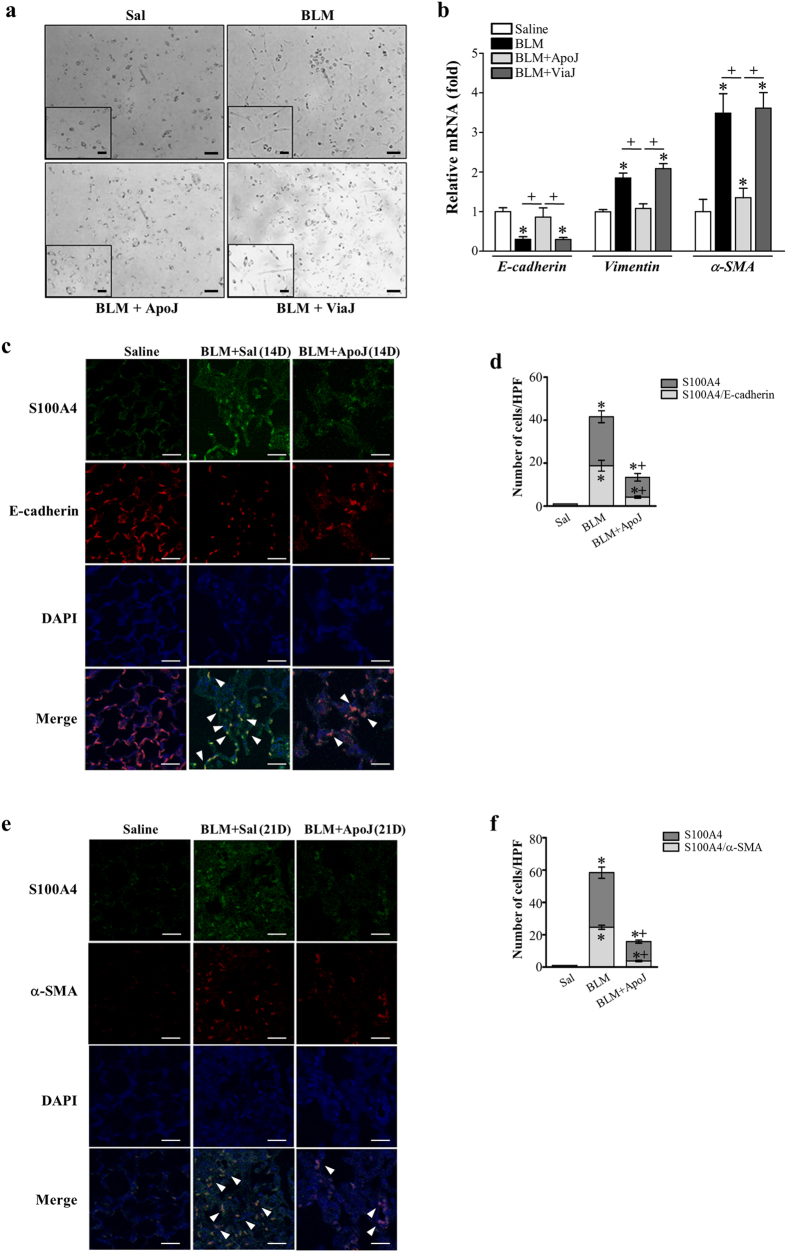
Reduction of EMT and fibrotic activation by *in vivo* instillation of apoptotic cells. Two days after bleomycin (BLM) treatment, lungs were instilled with saline alone (Sal), viable Jurkat cells (ViaJ), or apoptotic Jurkat cells (ApoJ) intratracheally. Mice were euthanized on days 14 or 21 following BLM treatment. Primary mouse alveolar type II epithelial (AT II) cells were isolated from murine lungs at 14 days after BLM treatment. The extent of EMT was determined by assessing the morphological changes (Scale bars = 100 μm) (**a**) and mRNA expression profiles of *E-cadherin, vimentin,* and *α-SMA* (**b**). The mRNA abundance of these EMT markers was analyzed by real-time PCR in primary AT II cells from each group on day 14 after BLM treatment. Values represent the mean ± s.e.m. from five mice in each group. *P < 0.05 compared with control; ^+^*P* < 0.05 as indicated. Immunofluorescence staining for E-cadherin (*red*), α-SMA (*red*), or fibroblast-specific protein-1 (S100A4, *green*) was performed in lung sections on days 14 (**c**) or 21 (**e**) following BLM treatment. Arrowheads indicate co-localization of E-cadherin or α-SMA in lung fibroblasts. The imaging medium was Vectashield fluorescence mounting medium containing DAPI. (**c**,**e**) Scale bars = 20 μm. Representative images were obtained from three mice in each group. Graph representing the number of double positive cells of S100A4 and E-cadherin or α-SMA compared with the total S100A4 positive cell population in lung parenchyma on days 14 (**d**) or 21 (**f**) after bleomycin treatment. Mean of 5 high power fields per section ± s.e.m. from three mice in each group. *P < 0.05 compared with control; ^+^*P* < 0.05 for BLM + ApoJ vs. BLM + Sal.

Next, we examined the effect of apoptotic cells on EMT in the bleomycin-induced lung injury murine model. At 14 d following bleomycin treatment, primary AT II cells from mice exposed to apoptotic Jurkat cells changed morphology from an elongated fibroblast-like shape to a typical rounded shape (Fig. 8a), and reduced *E-cadherin* mRNA and enhanced mesenchymal markers, such as *vimentin* and *α-SMA,* were reversed (Fig. 8b). However, instillation of viable Jurkat cells had no effect on EMT markers or morphology.

To confirm the EMT inhibitory effect of apoptotic cell instillation in alveolar epithelial cells after bleomycin treatment, we performed double immunofluorescence staining for α-SMA and fibroblast-specific protein-1 (FSP1) on lung tissue. E-cadherin/FSP1 double-positive cells, which reflect epithelial origin and a possible intermediate EMT-transition stage, were identified (Fig. 8c). Approximately 45% of the FSP1 positive fibroblasts were derived from lung epithelium 14 days after bleomycin treatment (Fig. 8d), indicating an apparent EMT phenomenon in mice following bleomycin treatment. Interestingly, both the number of epithelial-derived fibroblasts and FSP1 expression were reduced following apoptotic cell instillation (~26 % double-positive cells). Approximately 42% of the myofibroblasts expressing α-SMA in the interstitium co-localized with FSP1 (Fig. 8e,f). Apoptotic cell instillation decreased these markers and the number of double-positive cells in lung sections at 21 days after bleomycin treatment (~24% double-positive cells). Taken together, these data provide *in vivo* evidence that apoptotic cell instillation can prevent EMT and fibroblast activation in murine bleomycin-induced pulmonary fibrosis.

Previously, we demonstrated that COX-2 and HGF mRNA expression in alveolar macrophages and PGE_2_, and HGF production in BAL fluid from mice exposed to apoptotic Jurkat cells persistently enhanced up to 21 days after bleomycin treatment^30^,^31^. In the present study, we also found PGD_2_ production in BAL fluid was enhanced following apoptotic cell instillation at 14 days after bleomycin treatment (Supplementary Fig. S10a). We further investigated the role of macrophages for anti-EMT effects induced by apoptotic cell instillation. PGE_2_, PGD_2_, and HGF levels in alveolar macrophages taken from the bleomycin + apoptotic cell group at 14 days after bleomycin treatment were greater than those from the group of bleomycin + saline or viable Jurkat cells (Supplementary Fig. S10b-d). These data suggest that alveolar macrophages play a critical role for production of bioactive molecules in response to *in vivo* exposure to apoptotic cells, mediating anti-EMT process in alveolar epithelial cells. To confirm that PGE_2_-, PGD_2_, or HGF pathway is involved in anti-EMT effects induced by apoptotic cell instillation, the EP4, EP2, DP1, or c-Met antagonist was co-administered with apoptotic cells into the bleomycin-stimulated lungs. The enhanced E-cadherin, and the reduced vimentin, FSP1 and α-SMA mRNA and protein abundance in lung tissue by apoptotic cell instillation on days 14 or 21 after bleomycin treatment was reversed by AH-6809, AH-23848, BW-A868C (Supplementary Fig. S10e,g), or PHA-665752 treatment (Supplementary Fig. S10f,h). However, EP2 (AH-6809) showed inhibitory effect only on the enhanced E-cadherin mRNA and protein abundance by apoptotic cell instillation at 14 days after bleomycin treatment (Supplementary Fig. S10e,g). These data strongly suggest that PGE_2_, PGD_2_, and HGF’s pathways could mediate anti-EMT effects induced by exposure to apoptotic cells *in vivo*.

## Discussion

Programming of macrophages by apoptotic cells may influence epithelial cell homeostasis during lung injury^28^,^42^. Recent studies provide evidence that TGF-β1-induced EMT of alveolar epithelial cells may contribute to the formation of myofibroblasts in murine fibrotic lungs and IPF patient lungs^43^,^44^,^45^,^46^,^47^,^48^. In the present study, we used *in vitro* models of TGF-β1-induced EMT in alveolar epithelial cells to determine whether interaction of phagocytes with apoptotic cells results in an anti-EMT effect. We first demonstrated that ApoJ-exposed CM inhibited TGF-β1-induced EMT in LA-4 cells and primary mouse AT II cells as well as HEK-293 kidney epithelial cells. This inhibition in LA-4 cells was also observed when RAW 264.7 cells were exposed to different apoptotic cell types, such as LA-4 epithelial cells or primary mouse thymocytes, indicating a universal phenomenon that is cell-type independent. The TGF-β1-induced EMT inhibition did not occur when LA-4 cells were incubated with viable-exposed or necrotic-exposed CM, indicating specificity for apoptotic cells. Importantly, this anti-EMT effect on LA-4 cells was confirmed using conditioned media derived from primary murine BMDM exposed to apoptotic Jurkat cells. Collectively, these data suggest that anti-EMT effects are primarily caused by trophic factors released by macrophages, acting in a paracrine manner on lung and kidney epithelial cells.

The expression of EMT core regulators, including Snail, Zeb, and Twist, is induced in response to TGF-β1 through both Smad-dependent and -independent mechanisms^49^. Upon activation, these transcription factors repress epithelial marker gene expression and concomitantly activate mesenchymal gene expression. We demonstrated that ApoJ-exposed CM, but not NecJ-exposed CM, substantially suppressed TGF-β1-induced mRNA expression of *Snail1/2, Zeb1/2,* and *Twist1* in LA-4 cells. We further demonstrated that biological mediators in the ApoJ-exposed CM antagonize TGF-β1 signaling by partially blocking intracellular Smad-independent signaling pathways, such as p38 MAP kinase and PI3k/Akt pathways, but not Smad-dependent signaling pathways. These data indicate that downregulation of Smad-independent signaling leads to the inactivation of transcription factors that bind to *Snail1/2, Zeb1/2, and Twist1* promoters. However, because the inhibition of the p38 MAP kinase and PI3k/Akt pathways was not complete, another pathway may also be involved in controlling TGF-β1-induced EMT progression in LA-4 cells

Prostaglandins, including PGE_2_, PGD_2_, PGI_2_, PGF_2α_, and thromboxane A_2_ (TXA_2_), are derived from arachidonic acid by constitutive COX-1 and inducible COX-2. Interestingly, the PGE_2_, PGD_2_, and PGI_2_ synthesis levels are enhanced in RAW 264.7 cells in response to apoptotic cells^14^. Data from our previous and present studies confirmed that COX-2-derived PGE_2_ and PGD_2_ are released from RAW 264.7 cells in response to apoptotic cells^40^. In addition, RAW 264.7 and primary peritoneal macrophages induce HGF production via RhoA/Rho kinase-dependent signaling in response to apoptotic cells^41^. While somewhat controversial, COX-2/PGE_2_ and HGF were demonstrated to be negative modulators of fibrotic remodeling-associated EMT^32^,^50^,^51^. Thus, we focused on demonstrating the role of these molecules in anti-EMT effects induced by ApoJ-exposed CM. Both pharmacologic inhibition of COX-2 activity and siRNA-mediated knockdown of COX-2 expression in RAW 264.7 cells^40^ reversed the reduction of TGF-β1-induced EMT by ApoJ-exposed CM in LA-4 cells. However, knockdown of COX-1 expression did not influence the anti-EMT effect induced by ApoJ-exposed CM. These findings suggest that COX-2, but not COX-1, is specifically involved in the anti-EMT effect induced by the interaction of macrophages with apoptotic cells.

Treatment with exogenous PGE_2_ and PGD_2_, but not other prostaglandins, including PGI_2_, PGF_2α_, and TXA_2_, has been demonstrated to inhibit TGF-β-induced EMT in renal epithelial cells^32^,^39^. Moreover, exogenous PGE_2_ has been shown to suppress TGF-β-induced fibronectin and type 1 collagen α2 (Col1A2) expression in human lung cancer A549 cells^50^. Considering the effect of EP1/2/3/4 agonists, the suppressive effect of PGE_2_ on TGF-β1-induced EMT may be mediated through the EP2 and EP4 receptors^50^. In our current study, the EP2 antagonist AH-6809 only partially reversed the reduction of TGF-β1-induced EMT in LA-4 cells, whereas the antagonists of EP4, DP1, and DP2 reversed the effects on mesenchymal and epithelial markers as well as the transcriptional modulators *Snail1/2, Zeb1/2,* and *Twist1*. The importance of the PGE_2_-EP4 axis in limiting TGF-β1-induced EMT has also been demonstrated using renal tubular epithelial cells isolated from wild-type and EP4^−/−^ mice^52^.

Previously, we reported that the interaction of apoptotic cells with macrophages upregulates COX-2/PGE_2_ and HGF expression via a positive feedback loop *in vitro* and *in vivo*^40^,^53^. Interestingly, a COX-2-specific inhibitor, SC-59635, partially blocked the restoration of the epithelial phenotype, E-cadherin expression, and inhibition of α-SMA expression by exogenous HGF, indicating that COX-2 mediates the action of HGF^32^. Here, we demonstrated that blocking HGF production in macrophages using a Rho kinase inhibitor (Y-27632) or RhoA siRNA, or disrupting endogenous HGF signaling in LA-4 cells using a c-Met inhibitor (PHA-665752) strongly inhibited the reduction of TGF-β1-induced EMT markers by the conditioned medium. Different molecular mechanisms for HGF’s interference with EMT have been identified in different cell models. In renal tubular epithelial cells, HGF was shown to upregulate expression of the Smad co-repressor SnoN, which interacts with the translocated Smad complex and blocks the expression of Smad-dependent genes including integrin-linked kinase^33^,^35^. However, upregulation of SnoN protein levels by HGF was not observed in rat lung epithelial cells^34^. Instead, HGF mediated the upregulation of Smad7, an inhibitor of TGF-β signaling, in an ERK1/2-dependent manner. We confirmed that COX-2-derived PGE_2_ and PGD_2_ and HGF from primary murine BMDM exposed to apoptotic Jurkat cells acts in a paracrine manner to reduce TGF-β1-induced EMT in LA-4 cells via their receptors, particularly EP4, DP1, DP2, and c-Met. Moreover, exogenous PGE_2_, PGD_2_, or HGF at the stimulated concentrations of 150, 17, and 400 pg/ml, respectively, reduced TGF-β1-induced EMT in LA-4 cells^40^,^41^. Interestingly, addition of all these mediators together at the stimulation concentrations to LA-4 cells did not result in synergistic effects. These findings support the concept that they may have the same target molecules to inhibit TGF-β1-induced signaling pathway. Indeed, in consistent with ApoJ-exposed CM, exogenous PGE_2_, PGD_2_, or HGF blocked Smad-independent TGF-β1 signaling, including the p38 MAP kinase and Akt pathways, but did not affect Smad-dependent TGF-β1 signaling.

The rodent bleomycin model of lung fibrosis allows the use of molecular tools to dissect the cellular and subcellular processes leading to fibrosis^54^. Our previous data emphasize that *in vivo* exposure to apoptotic cells has an anti-fibrotic effect, as assessed using lung hydroxyproline measurements and trichrome staining in the bleomycin-induced murine model^31^,^32^. In addition, we also demonstrated the reduction of fibroproliferation matrix markers, such as Col1A2 and fibronectin, and α-SMA protein in lung tissue following apoptotic cell instillation at days 14 and 21 after bleomycin treatment^53^. Our previous and present data demonstrated gradual enhancement of PGE_2_, PGD_2_, and HGF production in BAL fluid following apoptotic cells after bleomycin treatment^30^,^31^. In the present study, consistent with *in vitro* findings, PGE_2_, PGD_2_, and HGF levels were further enhanced in alveolar macrophages following *in vivo* exposure to apoptotic cells at 14 days after bleomycin treatment. These data suggest that alveolar macrophages are actively involved in anti-EMT process under *in vivo* condition. Furthermore, we found that apoptotic cell instillation in isolated AT II cells from mouse lungs 14 days after bleomycin treatment inhibited the EMT process, including morphological changes and mRNA expression of EMT markers, such as decreased *E-cadherin* and increased *vimentin*, and *α-SMA*. Apoptotic cell instillation may alter the induction of EMT and the differentiation capacity of lung fibroblasts in the alveolar interstitial space following bleomycin treatment, because immunofluorescence staining indicates reduced numbers of E-cadherin/FSP1 and α-SMA/FSP1 double-positive cells following apoptotic cell instillation. In consistent with *in vitro* findings, inhibition of PGE_2_, PGD_2_, and HGF’s pathways by co-administration of their specific receptor antagonists could also reverse the enhanced E-cadherin, and the reduced vimentin, FSP1 and α-SMA mRNA and protein abundance in lung tissue by exposure to apoptotic cells *in vivo* on days 14 or 21 after bleomycin treatment. Taken together, these data provide *in vivo* evidence that enhanced PGE_2_, PGD_2_, and HGF secretion in alveolar macrophages following apoptotic cell instillation protects against the EMT phenotype in AT II cells and fibroblast activation in murine bleomycin-induced lung fibrosis.

In summary, we have elucidated a novel role for macrophage interaction with apoptotic cells in antagonizing the TGF-β1-induced EMT process via the signaling of multiple secreted bioactive mediators, including the COX-2-derived PGE_2_ and PGD_2_ and RhoA-dependent HGF. In addition, this study provides new insights into a possible mechanism for the anti-fibrotic effect of apoptotic cell instillation after bleomycin treatment. This effect is due to apoptotic cells’ interference with EMT in alveolar epithelial cells, which would otherwise lead to fibroblast activation and ECM deposition. Considering that EMT is a major event in the pathogenesis of IPF and other fibrotic diseases in multiple organs, including the kidney, liver, and peritoneum, our data suggest that apoptotic cells could be used to develop effective new cell therapies to limit pathologic fibrosis in diverse organ diseases.

## Materials and Methods

### Reagents

NS-398, AH-23848, BW-A868C, BAY-u3405, PD-146176, PGE_2_, PGD_2_, and HGF were purchased from Cayman Chemical (Ann Arbor, MI, USA). AH-6809, Y-27632, bleomycin (Sigma-Aldrich Chemical Co., St. Louis, MO), and PHA-665752 (Santa Cruz Biotechnology, Santa Cruz, CA, USA) were used as supplied. The gene-specific relative RT-PCR kit was obtained from Invitrogen (Carlsbad, CA, USA), and M-MLV reverse transcriptase was purchased from Enzynomics (Hanam, Korea). The enzyme immunoassay (EIA) kits for PGD_2_ were obtained from Assay Designs (Ann Arbor, MI, USA). The antibodies used in this study were against E-cadherin (Abcam, Cambrige, MA, USA), N-cadherin (Cell Signaling Technology, Beverly, MA, USA), α-SMA, (Abcam), and β-actin (Sigma-Aldrich).

### Cell lines, culture, and stimulation

Murine RAW 264.7 macrophages (American Type Culture Collection (ATCC), Manassas, VA) were plated at a density of 10^5^ cells/ml and incubated overnight in Dulbecco’s modified Eagle’s medium (DMEM, Media Tech Inc., Washington, DC, USA) supplemented with 10% fetal bovine serum (FBS), 2 mM L-glutamine,100 U/ml penicillin, and 100 μg/ml streptomycin at 37 °C and 5% CO_2_. Prior to stimulation, the medium was replaced with serum-free *X-VIVO* medium (Lonza, Basel, Switzerland). The human leukemia T cell line, Jurkat, was obtained from ATCC and cultured in RPMI 1640 (Media Tech Inc.) containing 10% FBS. LA-4 cells were purchased from ATCC and grown in F12K medium (Lonza, Basel, Switzerland) containing 15% heat-inactivated FBS at 37 °C in 5% CO_2_.

### Isolation and Culture of Primary Cells

BMDM were differentiated from bone marrow myeloid stem cells of C57BL/6 mice as described previously^55^. After 7–10 days in culture with L929 complement DMEM, BMDM differentiation was confirmed by FACS analysis using anti-CD11b. Individual thymocytes were isolated from 3- to 4-week-old BALB/c mice by mincing the thymus through a 70-μm pore size cell strainer (BD Biosciences, Bedford, MA, USA). Primary murine AT II cells were isolated from BALB/c mice and purified using a modification of published methods^56^,^57^. The purity of AT II cells was typically > 90%, as assessed using pro-SP-C immunofluorescence staining.

### Induction of cell death

Human Jurkat T lymphocytes, LA-4 lung epithelial cells, and murine thymocytes were exposed to ultraviolet irradiation at 254 nm for 10 min followed by incubation in RPMI-1640 with 10% FBS for 2 h at 37 °C and 5% CO_2._ Evaluation of nuclear morphology using light microscopy on Wright-Giemsa-stained samples indicated that the irradiated cells were approximately 70–80% apoptotic^40^. Lysed (necrotic) Jurkat T cells were obtained by multiple freeze-thaw cycles^38^. Apoptosis and necrosis were confirmed by Annexin V-FITC/propidium iodide (BD Biosciences, San Jose, CA) staining followed by flow cytometric analysis on a FACSCalibur system (BD Biosciences)^28^.

### Co-incubation of macrophages with apoptotic, viable, or necrotic cells

Macrophages (10^5^ cells/ml) were incubated with Jurkat cells, LA-4 cells, or thymocytes (apoptotic, viable, or necrotic; 3 × 10^5^ cells/ml) for 20 h in serum-free media. The conditioned medium was harvested by centrifugation and filtration then used for EMT assays.

### Transient transfections

RAW 264.7 cells were transiently transfected with 1 μg/ml of siRNA specifically targeting either COX-2, COX-1, or RhoA or with control siRNA (Bioneer, Seoul, Korea) using 5 μl of siRNA transfection reagent (Genlantis, San Diego, CA, USA) according to the manufacturer’s protocol. The sequences used for COX-2 knockdown were as follows: sense, 5′-CUA UGA UAG GAG CAU GUA A-3′; and antisense, 5′-UUA CAU GCU CCU AUC AUA G-3′. The sequences used for COX-1 knockdown were as follows: sense, 5′-GAG GUA GGA ACU UUG ACU A-3′; and antisense, 5′-UAG UCA AAG UUC CUA CCU C-3′. The sequences used for RhoA knockdown were as follows: sense, 5′-GAA GUC AAG CAU UUC UGU CTT A-3′; and antisense, 5′- GAC AGA AAU GCU UGA CUU CTT-3′. The sequences for control siRNA were as follows: sense, 5′-CCU ACG CCA CCA AUU UCG U-3′; and antisense, 5′-ACG AAA UUG GUG GCG UAG G-3′. Cells were incubated in serum-free medium for 6 h for COX-2 siRNA, 48 h for COX-1 siRNA, or 24 h for RhoA siRNA prior to experimentation. None of the siRNAs used had any significant effect on cell viability.

### Incubation of epithelial cells with conditioned medium

LA-4 and HEK-293 cells were plated in 6-well culture plates (2 × 10^5^ cells/well) and cultured overnight in 200 μl RPMI 1640 or DMEM, respectively, containing 10% FBS. Primary AT II cells were plated and cultured on type 1 collagen-coated culture plates (1 × 10^6^ cells/well) for 48 h. Cells were treated for 24–72 h with conditioned medium from macrophages in the presence of 10 ng/ml TGF-β1^28^. In some experiments, 10 μM AH-6809, AH-23848, BW-A868C, or BAY-u3405 or 250 nM PHA-665752 was used to antagonize EP2, EP4, DP1, DP2, or c-Met, respectively. The antagonist was added 1 h before the addition of the conditioned medium with 10 ng/ml TGF-β1.

### Immunoblot analysis

To detect the expression of epithelial and mesenchymal markers, cells were lysed in 0.5% Triton X-100-containing lysis buffer and resolved on a 10% SDS-PAGE gel prior to transfer onto nitrocellulose. Membranes were blocked at room temperature with Tris-buffered saline containing 3% BSA and then incubated at room temperature with various anti-mouse primary antibodies and probed with mouse anti-mouse HRP-conjugated secondary antibody. Bands were visualized using enhanced chemiluminescence.

### Enzyme-linked immunosorbent assay (ELISA) measurement

Culture supernatants were collected and PGD_2_ concentration was measured using an EIA kit according to the manufacturer’s instructions.

### Quantitative real-time PCR (qPCR)

Gene expression was analyzed by real-time qPCR on a StepOnePlus system (Applied Biosystems, Life Technologies, Carlsbad, CA, USA). For each qPCR assay, a total of 50 ng cDNA was used. Primer sets for PCR-based amplifications were designed using Primer Express software. The primers used were as follows (name: forward primer, reverse primer). For mice, *E-cadherin*: 5′-GCACTCTTCTCCTGGTCCTG-3′, 5′-TATGAGGCTGTGGGTTCCTC-3′; *N-cadherin*: 5′-CCTCCAGAGTTTACTGCCATGAC-3′, 5′-CCACCACTGATTCTGTATGCCG-3′; *α-SMA*: 5′-CCACCGCAAATGCTTCTAAGT-3′, 5′-GGCAGGAATGATTTGGAAAGG-3′; *vimentin*: 5′-GCGTGCGGCTGCTTCAAGAC-3′, 5′- ATGGCGTCGGCCAGCGAGAA-3′; *FSP1*: 5′- GAAGTGCATTCCAGAAGGTGA-3′, 5′-CATCATGGCAATGCAGGACA-3′; *Snail1*: 5′-CCCAAGGCCGTAGAGCTGA-3′, 5′-GCTTTTGCCACTGTCCTCATC-3′; *Snail2*: 5′-ATCCTCACCTCGGGAGCATA-3′, 5′-TGCCGACGATGTCCATACAG-3′; *Zeb1*: 5′-ATTCAGCTACTGTGAGCCCTGC-3′, 5′-CATTCTGGTCCTCCACAGTGGA-3′; *Zeb2*: 5′-GCAGTGAGCATCGAAGAGTACC-3′, 5′-GGCAAAAGCATCTGGAGTTCCAG-3′; *Twist1*: 5′-TCGACTTCCTGTACCAGGTCCT-3′, 5′-CCATCTTGGAGTCCAGCTCG-3′; *HPRT*: 5′-CCAGTGTCAATTATATCTTCAAC-3′, 5′-CAGACTGAAGAGCTACTGTAATG-3′. The cDNA abundances were normalized to that of *glyceraldehyde 3-phosphate dehydrogenase* cDNA^58^ and are presented as the fold-change in abundance compared to the appropriate controls.

### Immunocytochemistry

Cells were grown on coverslips and cultured for the indicated time and conditions, followed by fixation with 2% formaldehyde (formalin) for 10 min. Cells were permeabilized with 0.1% Triton X-100 (Sigma-Aldrich) and stained with goat antibody against mouse E-cadherin (Abcam), α-SMA (Abcam), or goat IgG isotype control (all from Jackson ImmunoResearch Laboratories, West Grove, PA, USA). Cells were stained with rabbit polyclonal anti E-cadherin antibody (1:250 dilution) and mouse monoclonal anti-α-SMA antibody (1:250 dilution). Subsequently, cells were incubated with fluorescein isothiocyanate (FITC)-conjugated donkey anti-rabbit IgG (1:400; Jackson ImmunoReseach) and Alexa Fluor 546–conjugated goat anti-rabbit IgG (Molecular Probes, Life Technologies). The slides were mounted with Vectashield Mounting Medium containing DAPI (Vector Laboratories, Inc., Burlingame, CA, USA). All slides were imaged with a confocal microscope (LSM5 PASCAL, Carl Zeiss).

Quantification of elongated cell morphology. Morphological changes in the cells examined by phase-contrast microscopy were quantitated by measuring the lengths of the major and minor cell axes using Zeiss software (Zeiss LSM Image Browser^59^. The ratios of the major axis to the minor axis of cells were used to determine the degree of elongated cell morphology. For each experiment, at least 30 cells of each cell type were measured. Data were statistically analyzed using analysis of variance followed by Tukey’s post hoc test.

### Animal protocols

Specific pathogen-free male C57BL/6 mice (Orient Bio, Sungnam, Korea) weighing 20~25 g were used for all experiments. The Animal Care Committee of the Ewha Medical Research Institute approved the experimental protocol (N0. 11–0176). Mice were cared for and handled in accordance with the National Institute of Health (NIH) Guide for the Care and Use of Laboratory Animals. Mouse pharyngeal aspiration was used to administer a test solution containing bleomycin (5 U/kg body weight in 30 μl)^42^,^60^. Two days after bleomycin treatment, saline alone or 1 × 10^7^ apoptotic or viable Jurkat cells in 50 μl saline were administered intratracheally through pharyngeal aspiration^61^,^62^. Mice were euthanized on days 14 and 21 following bleomycin treatment.

For the inhibition experiments, the selective PGE_2_ receptor EP2 antagonist AH-6809 (5 mg/kg, i.p.)^31^, or EP4 antagonist AH-23848 (10 mg/kg, i.p.)^63^, or the selective PGD_2_ receptor DP1 antagonist BW-A868C (1 mg/kg, S.C.)^64^ or the selective c-Met inhibitor PHA-665752 (25 mg/kg, i.p.)^31^,^53^ was administrated at the same time as instillation of 10 × 10^6^ apoptotic Jurkat cells into bleomycin-stimulated lungs (2 days). After the first dose, the inhibitor was administrated once a day (AH-6809, AH-23848, and BW-A868C), and mice euthanized 14 days after bleomycin treatment. PHA-665752 (25 mg/kg) was administrated daily from days 10 to 20 after bleomycin treatment, and mice were euthanized 21 days following bleomycin treatment.

### Bronchoalveolar lavage (BAL) cells, lung tissue, and cell counts

BAL was performed through a tracheal cannula using 0.7 ml aliquots of ice-cold Ca^2+^/Mg^2+^-free phosphate-buffered medium (145 mM NaCl, 5 mM KCl, 1.9 mM NaH_2_PO_4_, 9.35 mM Na_2_HPO_4_, and 5.5 mM dextrose; pH 7.4) to a total of 3.5 ml for each mouse. BAL samples were centrifuged at 500 × *g* for 5 min at 4 °C, and cell pellets were washed and resuspended in phosphate-buffered medium. Cell counts were determined using an electronic Coulter Counter fitted with a cell sizing analyzer (Coulter Model ZBI with a channelizer 256; Coulter Electronics, Bedfordshire, UK). Alveolar macrophages were identified by their characteristic cell diameters. After BAL, lungs were removed, immediately frozen in liquid nitrogen, and stored at −70 °C.

### Preparation of alveolar macrophages

Alveolar macrophages were isolated as described previously, with slight modifications^16^,^32^. In brief, suspended alveolar macrophages were over 95% viable as determined by trypan blue dye exclusion. Alveolar macrophages (5 × 10^5^ per well in 12-well plates) were cultured in serum-free *X-vivo* medium for 60 min. Nonadherent cells were removed by washing three times before isolation of total RNA. Approximately 90–95% of the plastic-adherent cells were morphologically macrophages.

### Enzyme-linked immunosorbent assay (ELISA)

PGE_2_, PGD_2_, and HGF were measured from the 18 h supernatants of cultured alveolar macrophages or BAL fluid using a specific ELISA kit as per the manufacturer’s instructions (R&D Systems, Minneapolis, MN).

### Immunohistochemistry

Four-μm-thick sections were obtained from formalin-fixed, paraffin-embedded tissues. Slides were deparaffinized twice in xylene and rehydrated through graded ethanol solutions to distilled water. Sections were incubated with primary antibodies against E-cadherin, α-SMA, FSP1, or control rabbit IgG at room temperature for 30 min. Cells were incubated with Texas Red-conjugated anti-mouse secondary antibody and FITC-conjugated goat anti-rat secondary antibody (Vector Laboratories, Inc.). The sections were washed with Tris-buffered saline between all steps. The sections were mounted in Vectashield Mounting Medium with DAPI. All slides were imaged using a confocal microscope.

### Statistical analysis

Data are expressed as the mean ± S.E. Analysis of variance was used for multiple comparisons and Tukey’s post hoc test was used where appropriate. The Student’s *t-*test was used for comparing two sample means. A *P*-value less than 0.05 was considered statistically significant. All data were analyzed using JMP software (SAS Institute, Cary, NC).

## Additional Information

**How to cite this article**: Yoon, Y.-S. *et al.* Macrophages programmed by apoptotic cells inhibit epithelial-mesenchymal transition in lung alveolar epithelial cells via PGE_2_, PGD_2_, and HGF. *Sci. Rep.* **6**, 20992; doi: 10.1038/srep20992 (2016).

## Supplementary Material

Supplementary Information
