# Supplementary material for: Macrophages programmed by apoptotic cells inhibit epithelial-mesenchymal transition in lung alveolar epithelial cells via PGE2, PGD2, and HGF
Source: Sci Rep. 2016 Feb 15;6:20992. doi: 10.1038/srep20992 (PMC4753481; doi:10.1038/srep20992)
Supplement: Supplementary Information [file srep20992-s1.pdf]

Supplementary Information

Macrophages programmed by apoptotic cells inhibit epithelial-mesenchymal transition in lung alveolar epithelial cells via PGE_2, PGD_2, and HGF

Young-So Yoon[1,2], Ye-Ji Lee[1,2], Youn-Hee Choi[1,2], Young Mi Park[3] & Jihee Lee Kang[1,2*]

[1]Department of Physiology, School of Medicine, Ewha Womans University, Seoul 158-710, Korea
[2]Tissue Injury Defense Research Center, School of Medicine, Ewha Womans University, Seoul 158-710, Korea
[3]Department of Molecular medicine, School of Medicine, Ewha Womans University, Seoul 158-710, Korea

*Corresponding author: Dr. Jihee Lee Kang, Department of Physiology, Tissue Injury Defense Research Center, School of Medicine, Ewha Womans University, 911-1 Mok-6-dong, Yangcheon-ku, Seoul 158-056, Korea (Telephone) 82-2-2650-5719, (Fax) 82-2-2650-5717. E-mail: jihee@ewha.ac.kr

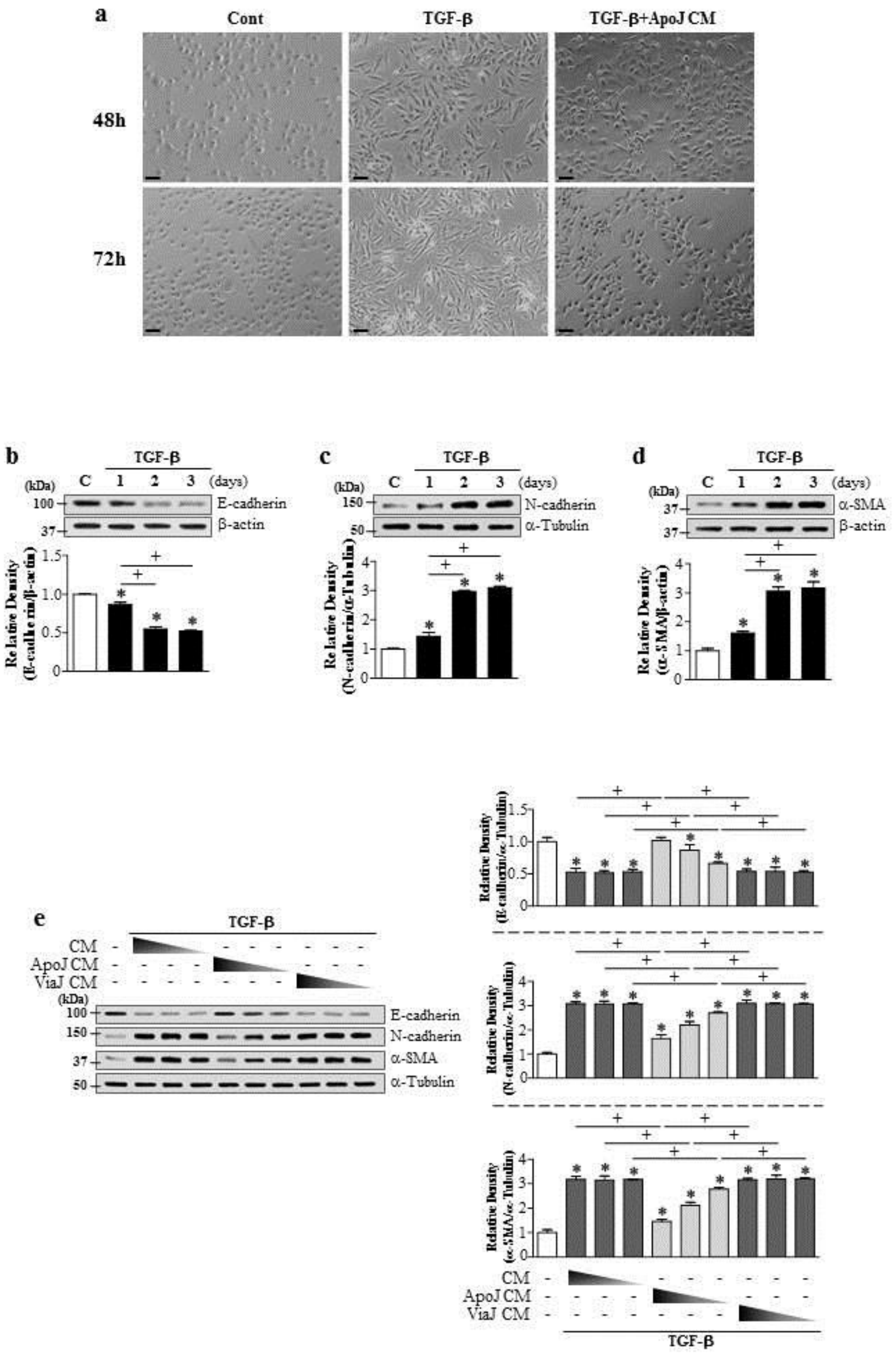

Supplementary Figure S1. Effects of conditioned medium from RAW 264.7 cells exposed to apoptotic Jurkat T cells on TGF-β-induced EMT in lung epithelial cells. RAW 264.7

cells were stimulated with apoptotic (ApoJ), or viable (ViaJ) Jurkat T cells for 20 h. Conditioned medium (CM) was added to LA-4 cells in the absence or presence of 10 ng/ml TGF-β1. (a) Morphological changes in the cells were examined by phase-contrast microscopic observation at 48 and 72 h after TGF-β1 treatment. Scale bars = 100 μm. Immunoblots of total cell lysates were performed with anti-E-cadherin, N-cadherin, or α-SMA antibodies at the indicated time points (b-d) or 72 h (e) after TGF-β1 treatment. (e) Effects of CM (no dilution, 1:1, or 1:3 dilution with X-vivo medium) from RAW 264.7 cells exposed to apoptotic on changes of abundance of EMT markers in lung epithelial cells at 72 h after treatment with TGF-β1. Densitometric analysis of the indicated EMT markers' relative abundances. Values represent the mean ± s.e.m. of three independent experiments. $^{*}P < 0.05$ compared with control; $^{+}P < 0.05$ as indicated.

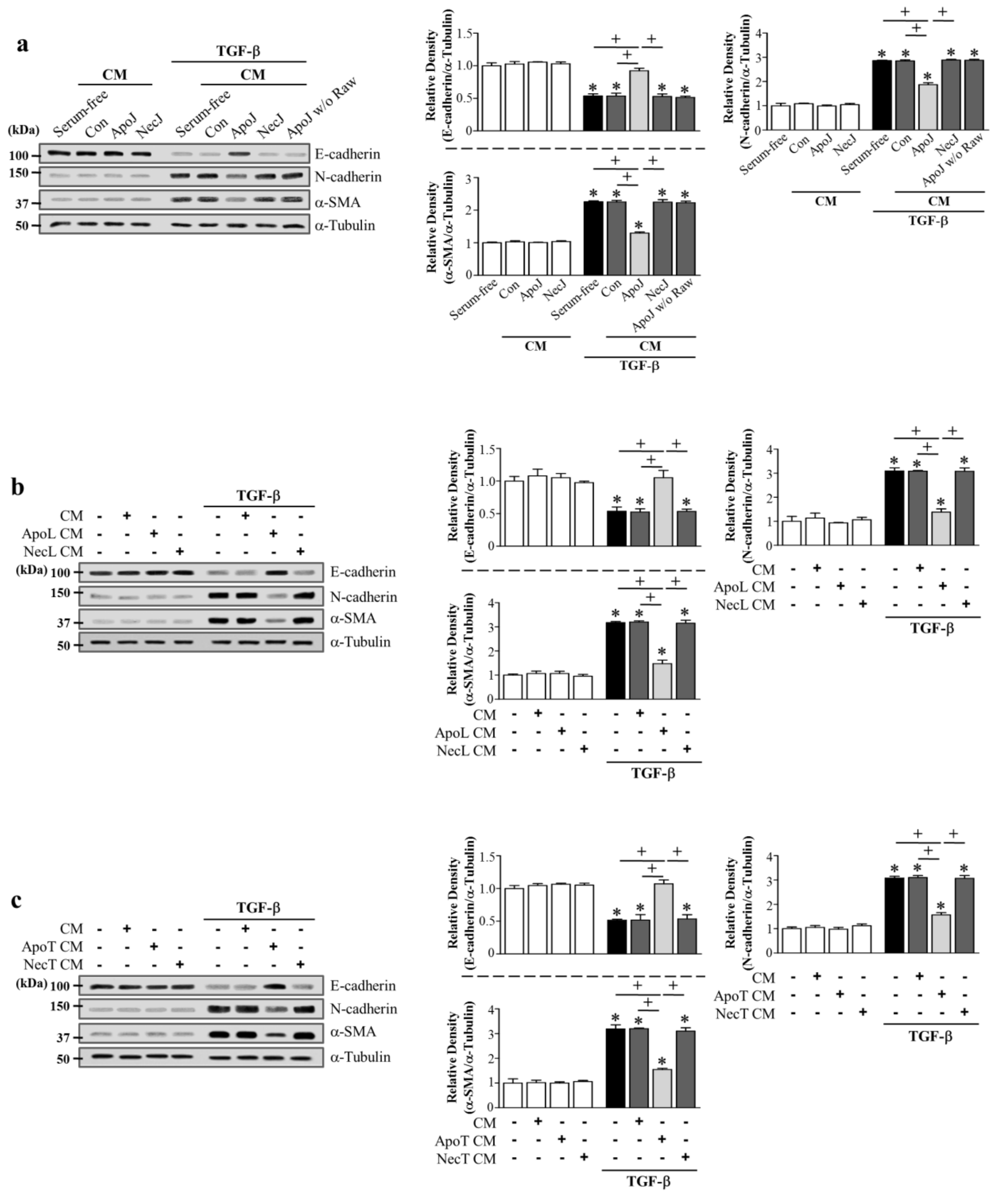

Supplementary Figure S2. Conditioned medium from RAW 264.7 cells exposed to apoptotic cells reduced TGF-β1-induced EMT in lung and kidney epithelial cells. RAW 264.7 cells were stimulated with apoptotic or necrotic cells of Jurkat cells (ApoJ, NecJ) (a) or LA-4 cells (ApoL, NecL) (b) or thymocytes (ApoT, NecT) (c) for 20 h. Conditioned medium (CM) was added to HEK-293 kidney epithelial cells (a) or LA-4 cells (b, c) in the absence or presence of 10 ng/ml TGF-β1. After 72 h, immunoblots of total cell lysates were performed

with anti-E-cadherin, N-cadherin, or α-SMA antibodies. Right: Densitometric analysis of the relative abundances of the indicated EMT markers. Values represent the mean ± s.e.m. of three independent experiments. *$P < 0.05$ compared with control; $^{+}P < 0.05$ as indicated.

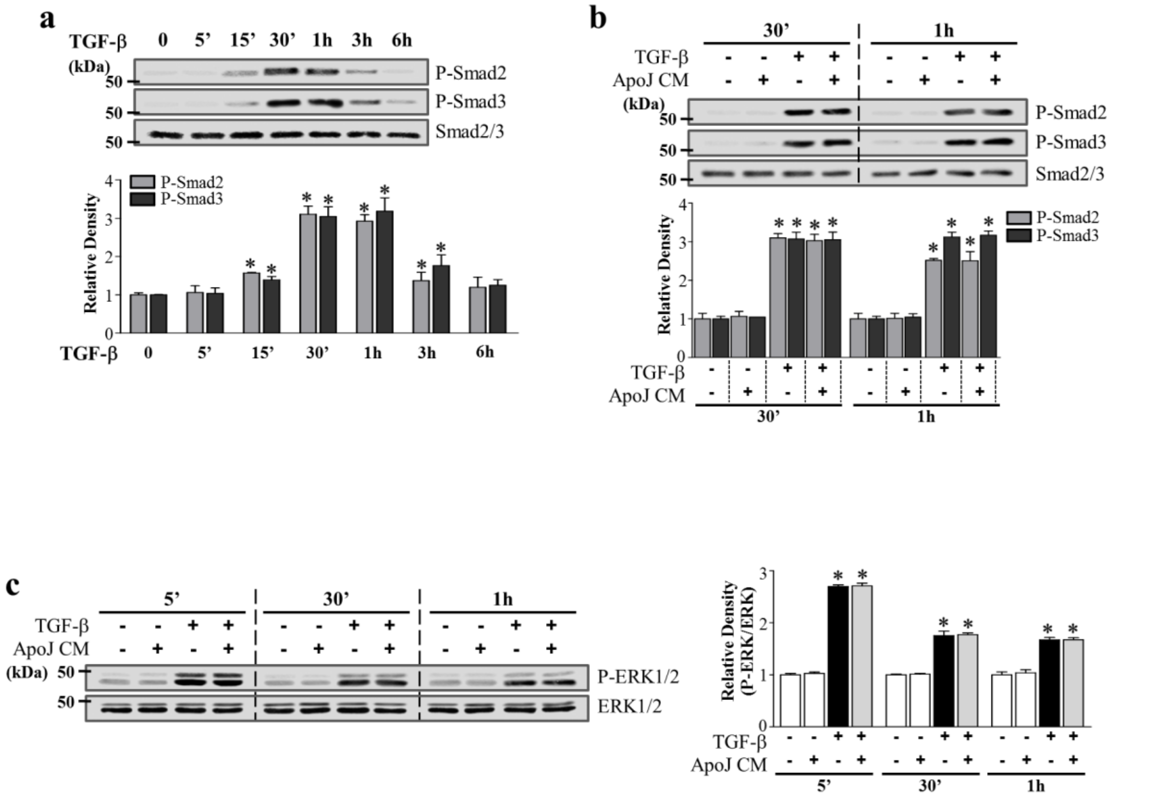

Figure 3. Conditioned medium from RAW 264.7 cells exposed to apoptotic cells did not block Smad-dependent and ERK-dependent TGF-β1 signaling in LA-4 cells. RAW 264.7 cells were stimulated with apoptotic Jurkat cells (ApoJ) for 20 h. Conditioned medium (CM) was added to LA-4 cells in the absence or presence of 10 ng/ml TGF-β1 for the indicated time. (a-c) Western blot analysis of the relative amounts of total and phosphorylated Smad2. Smad3, and ERK protein in the indicated samples over time. Densitometric analysis of the relative phosphorylated protein abundances, normalized to that of total protein. Data in all bar graphs are the mean ± s.e.m. of three independent experiments. *$P < 0.05$ compared with control.

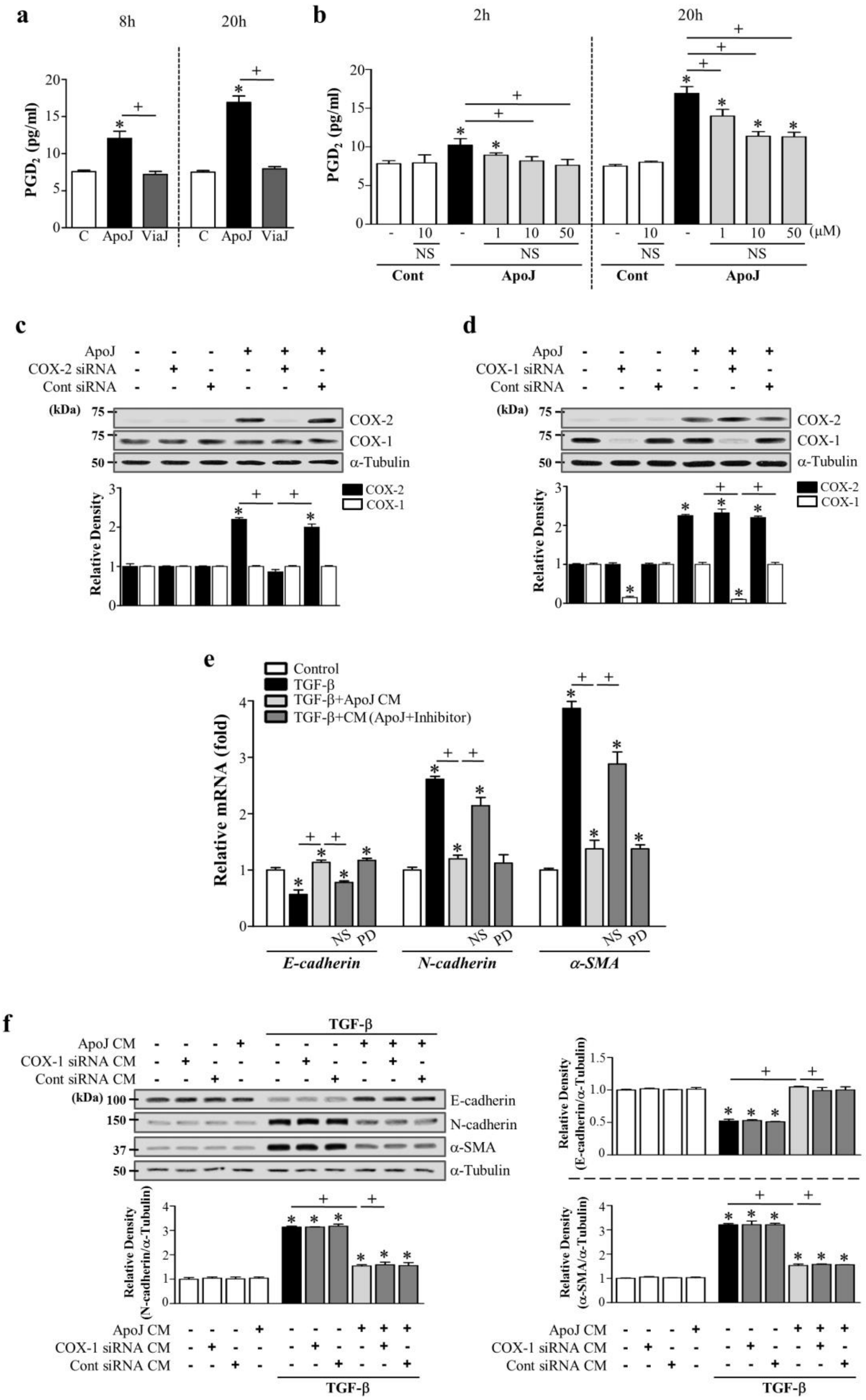

Supplementary Figure S4. Apoptotic cells induce PGD_2 by RAW 264.7 cells and COX-2 signaling mediates reduction of EMT process. (a) RAW 264.7 cells were stimulated by

apoptotic (ApoJ), or viable (ViaJ) Jurkat T cells for the times indicated. (b) After 1 h pretreatment with 1-50 μM of NS-398, RAW cells were stimulated with ApoJ for 2 or 20 h. PGD_2 levels in the conditioned medium (CM) were measured by EIA. (c) RAW cells were transfected with COX-2 or control vehicle (siRNA-GFP) for 6 h, then incubated with ApoJ for 20 h. (d, f) RAW cells were transfected with COX-1 siRNA or control vehicle (siRNA-GFP) for 48 h, then incubated with ApoJ for 20 h. (f) Conditioned medium (CM) was added to LA-4 cells in the presence of TGF-β1 for 72 h. (c, d, f) Immunoblots of total cell lysates were performed with anti-COX-2, COX-1, E-cadherin, N-cadherin, or α-SMA antibodies. Right: Densitometric analysis of the indicated EMT markers' relative abundances. (e) RAW 264.7 cells were pretreated with 10 μM NS-398 or 10 μM PD-146176 for 1 h and then stimulated with ApoJ for 20 h. After 72 h, the amounts of *E-cadherin, N-cadherin, or α-SMA* mRNAs in LA-4 cell samples were analyzed by real-time PCR and normalized to that of *Hprt* mRNA. Values represent the mean ± s.e.m. of three independent experiments. $^{*}P < 0.05$ compared with control; $^{+}P < 0.05$ as indicated.

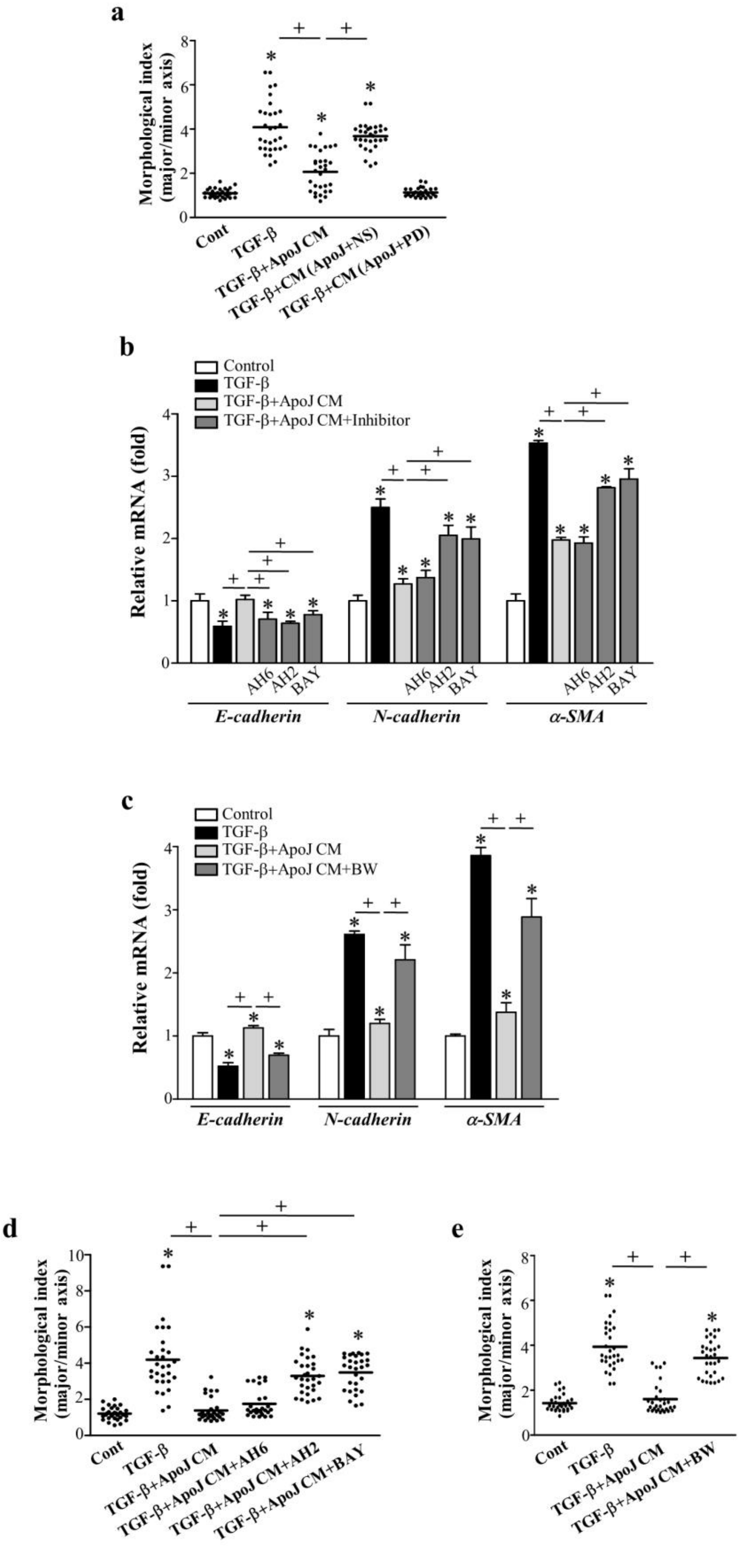

Supplementary Figure S5. COX-2 derived PGE_2 and PGD_2 from RAW 264.7 cells in

response to apoptotic cells mediate EMT inhibition in LA-4 cells via their receptors. (a) RAW 264.7 cells were pretreated with 10 μM NS-398 or 10 μM PD-146176 for 1 h and then stimulated with apoptotic Jurkat cells (ApoJ) for 20 h. Conditioned medium (CM) was added to LA-4 cells in the presence of TGF-β1 for 72 h. Quantitative analysis of cell morphology of TGF-β1-treated cells in Figure 4a (a) and 5a (d, e), showing the degree of elongated cell morphology, or morphological index. Horizontal lines indicate the mean value for each experimental group. Representative data shown are from a single experiment, for which at least 30 cells were examined for each experimental group. (b-e) RAW 264.7 cells were stimulated with ApoJ for 20 h. Conditioned medium (CM) was added to LA-4 cells in the presence of TGF-β1 with or without antagonists of EP2 (10 μM AH-6809), EP4 (10 μM AH-23848), DP1 (10 μM BW-A868C), or DP2 (10 μM BAY-u3405). After 48 (b) or 72 h (c), the amounts of *E-cadherin*, *N-cadherin*, *or α-SMA* mRNAs in LA-4 cell samples were analyzed by real-time PCR and normalized to that of *Hprt* mRNA. Values represent the mean ± s.e.m. of three independent experiments. $^{*}P < 0.05$ compared with control; $^{+}P < 0.05$ as indicated.

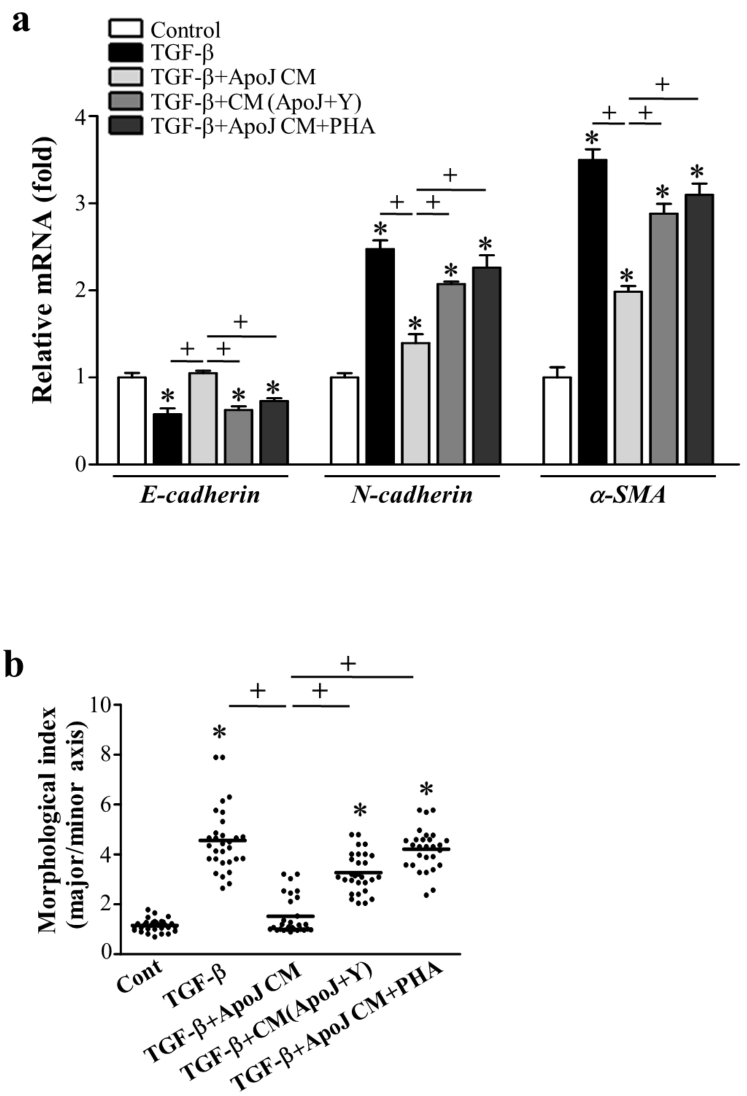

Supplementary Figure S6. Inhibition of RhoA in RAW 264.7 cells and HGF signaling in LA-4 cells reverse EMT reduction in LA-4 cells. RAW 264.7 cells stimulated with apoptotic Jurkat cells (ApoJ) with or without with 30 μM Y-27632, After 20 h, conditioned medium (CM) was added to LA-4 cells in the presence of TGF-β1 with or without the antagonist of c-Met (250 nM PHA-665752). (a) After 48 h, the amounts of *E-cadherin, N-cadherin, or α-SMA* mRNAs in LA-4 cell samples were analyzed by real-time PCR and normalized to that of *Hprt* mRNA. (b) Quantitative analysis of cell morphology of TGF-β1-treated cells in Figure 6a, showing the degree of elongated cell morphology, or morphological index. Horizontal lines indicate the mean value for each experimental group. Representative data shown are from a single experiment, for which at least 30 cells were examined for each experimental group. Values represent the mean ± s.e.m. of three independent experiments. *$P < 0.05$ compared with control; $^{+}P < 0.05$ as indicated.

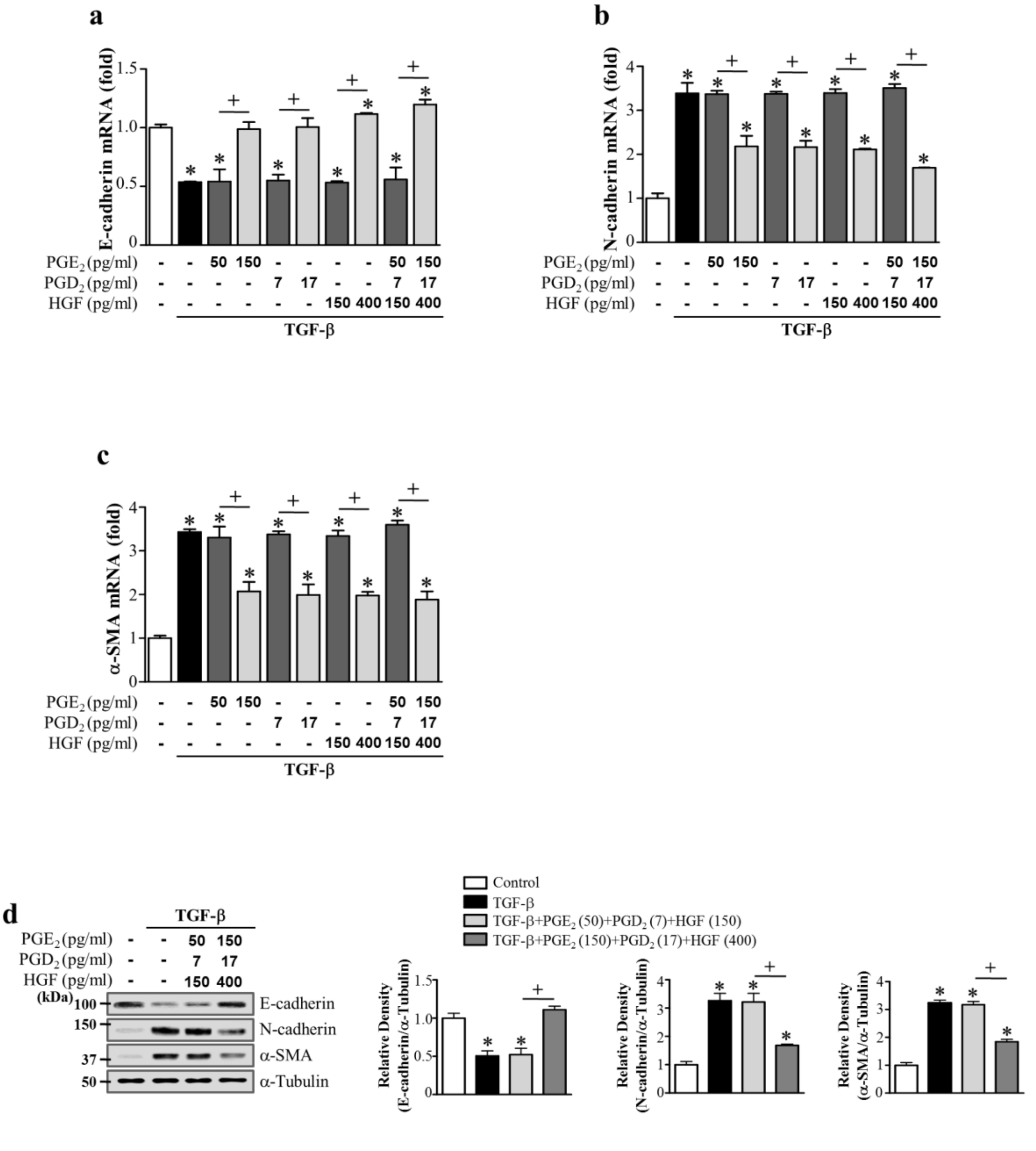

Supplementary Figure S7. Exogenous PGE_2, PGD_2, and HGF mediate EMT reduction in LA-4 cells. PGE_2 (50 and 150 pg/ml), PGD_2 (7 and 17 pg/ml), and HGF (150 and 400 pg/ml) were added individually or all together to LA-4 cell culture in the presence of TGF-β1 for 48 h. (a-c) The amount of EMT markers' mRNA in LA-4 cell samples was analyzed by real-time PCR and normalized to that of *Hprt* mRNA. (d) Immunoblots of total cell lysates were performed with anti-E-cadherin, anti-N-cadherin, or anti-α-SMA antibody in the indicated samples over time. Right: Densitometric analysis of the indicated EMT markers' relative abundances. Values represent the mean ± s.e.m. of three independent experiments. $^{*}P < 0.05$ compared with control; $^{+}P < 0.05$ as indicated.

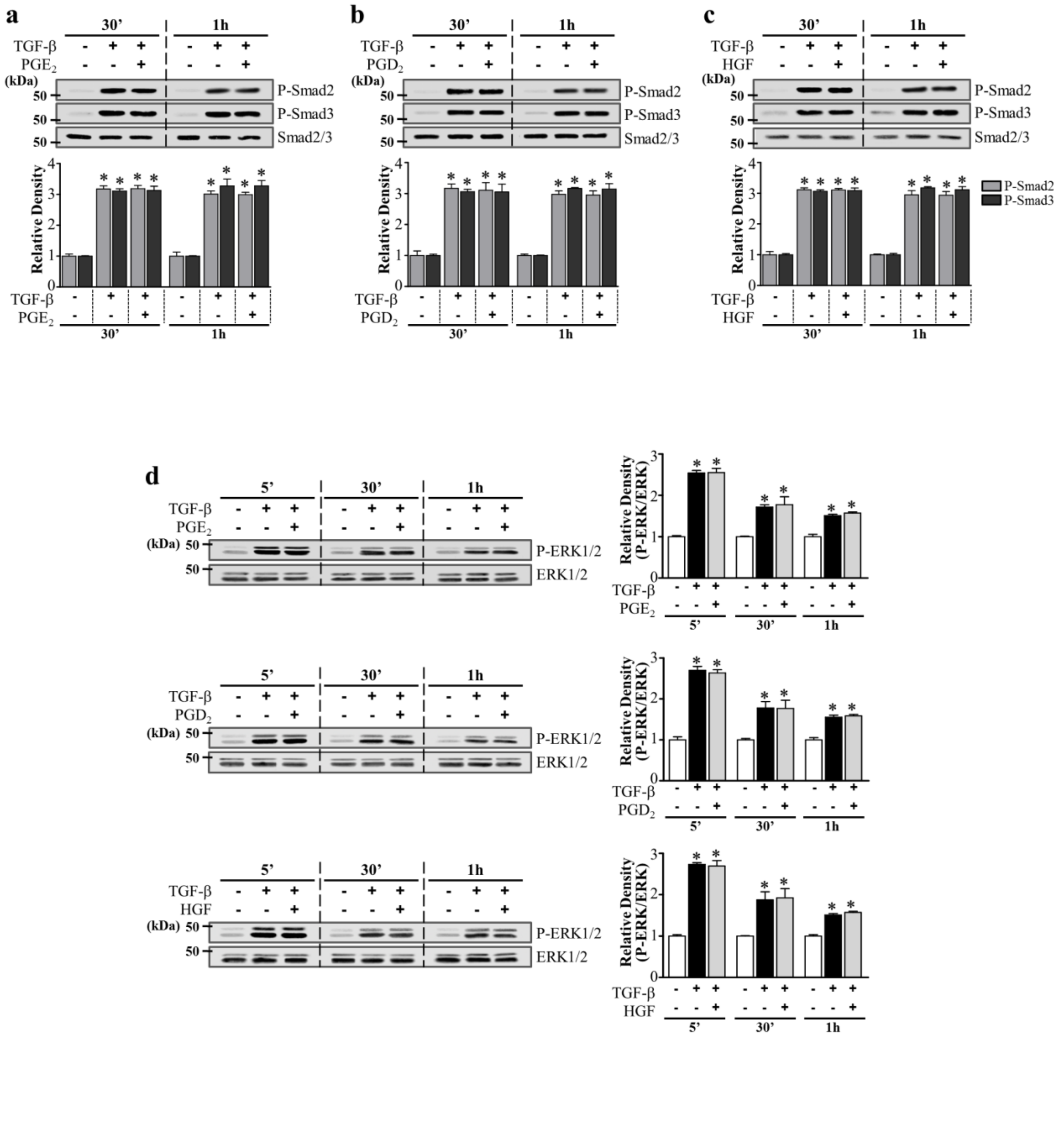
a
b
c
d
30’
1h
5’
TGF-β
PGE2
PGD2
HGF
(kDa)
50
P-Smad2
P-Smad3
Smad2/3
Relative Density
P-ERK1/2
ERK1/2
Relative Density (P-ERK/ERK)

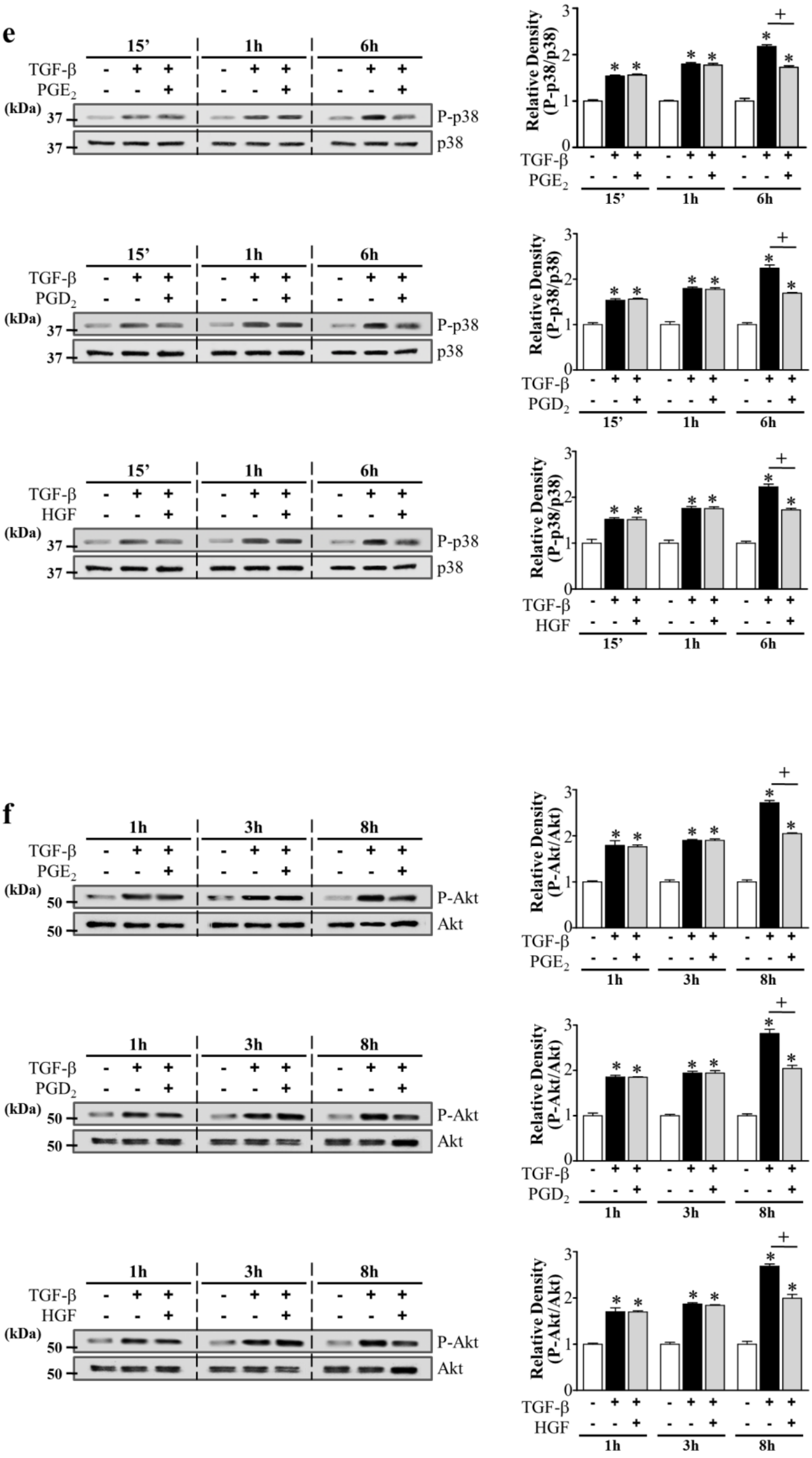

Supplementary Figure S8. Exogenous PGE_2, PGD_2, and HGF block Smad-independent TGF-β1 signaling in LA-4 cells. PGE_2 (50 and 150 pg/ml), PGD_2 (7 and 17 pg/ml), or HGF

(150 and 400 pg/ml) was added to LA-4 cell culture in the presence of TGF-β1 for 48 h. Immunoblots of total cell lysates were performed with anti- Smad2, anti-phospho-Smad2, anti-Smad3, anti-phospho-Smad3 (a-c), anti-ERK, anti-phospho-ERK (d), anti-p38 MAP kinase, anti-phospho-p38 MAP kinase(e), or anti-Akt, or anti-phospho-Akt antibody (f) in the indicated samples over time. Densitometric analysis of the indicated EMT markers' relative abundances. Values represent the mean ± s.e.m. of three independent experiments. $^{*}P < 0.05$ compared with control; $^{+}P < 0.05$ as indicated.

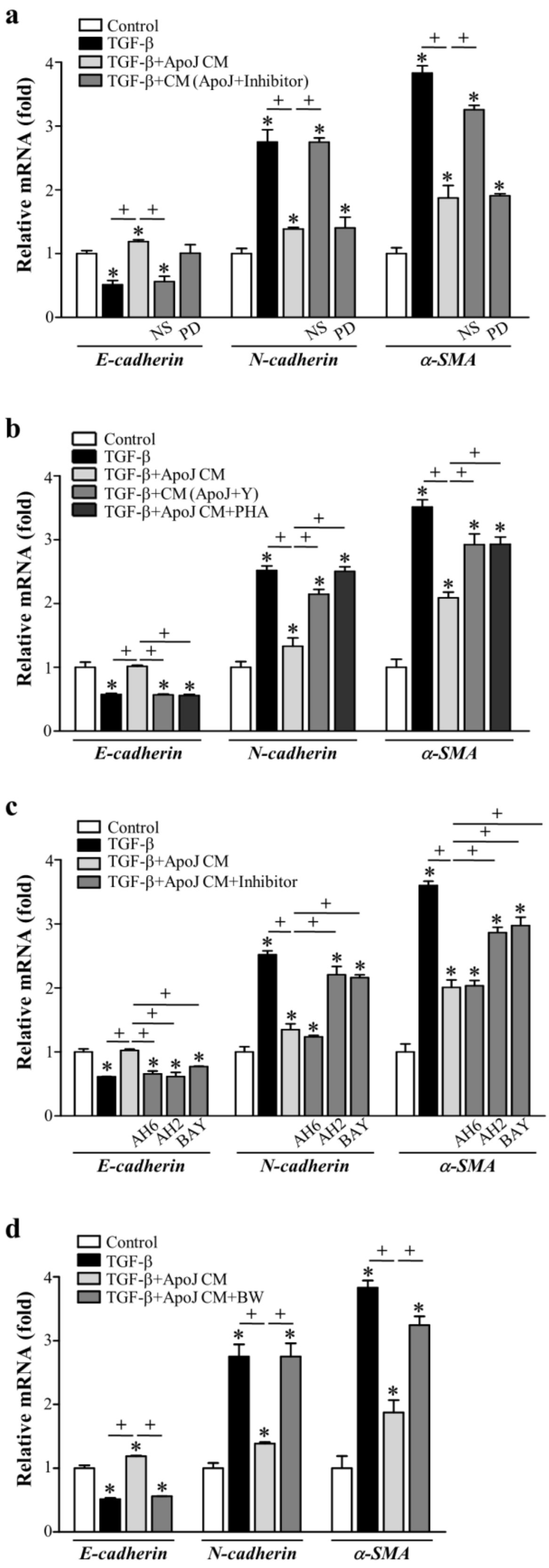

Supplementary Figure S9. PGE_2, PGD_2, and HGF from murine BMDM in response to apoptotic cells mediate EMT inhibition in LA-4 cells via their receptors. Murine BMDM

were pretreated with 10 μM NS-398, 30 μM PD-146176 (a), or 30 μM Y-27632 (b) for 1 h and then stimulated with apoptotic Jurkat cells (ApoJ) for 20 h. Conditioned medium (CM) was added to LA-4 cells in the presence of TGF-β1 for 48 (b) or 72 h (a). (c, d) Murine BMDM were stimulated with ApoJ for 20 h. CM was added to LA-4 cells in the presence of TGF-β1, with or without antagonists of EP2 (10 μM AH-6809), EP4 (10 μM AH-23848), DP1 (10 μM BW-A868C), DP2 (10 μM BAY-u3405), or c-Met (250 nM PHA-665752) for 48 (b, c) or 72 h (d). The amounts of *E-cadherin*, *N-cadherin*, or *α-SMA* mRNAs in LA-4 cell samples were analyzed by real-time PCR and normalized to that of *Hprt* mRNA. Values represent the mean ± s.e.m. of three independent experiments. $^{*}P < 0.05$ compared with control; $^{+}P < 0.05$ as indicated.

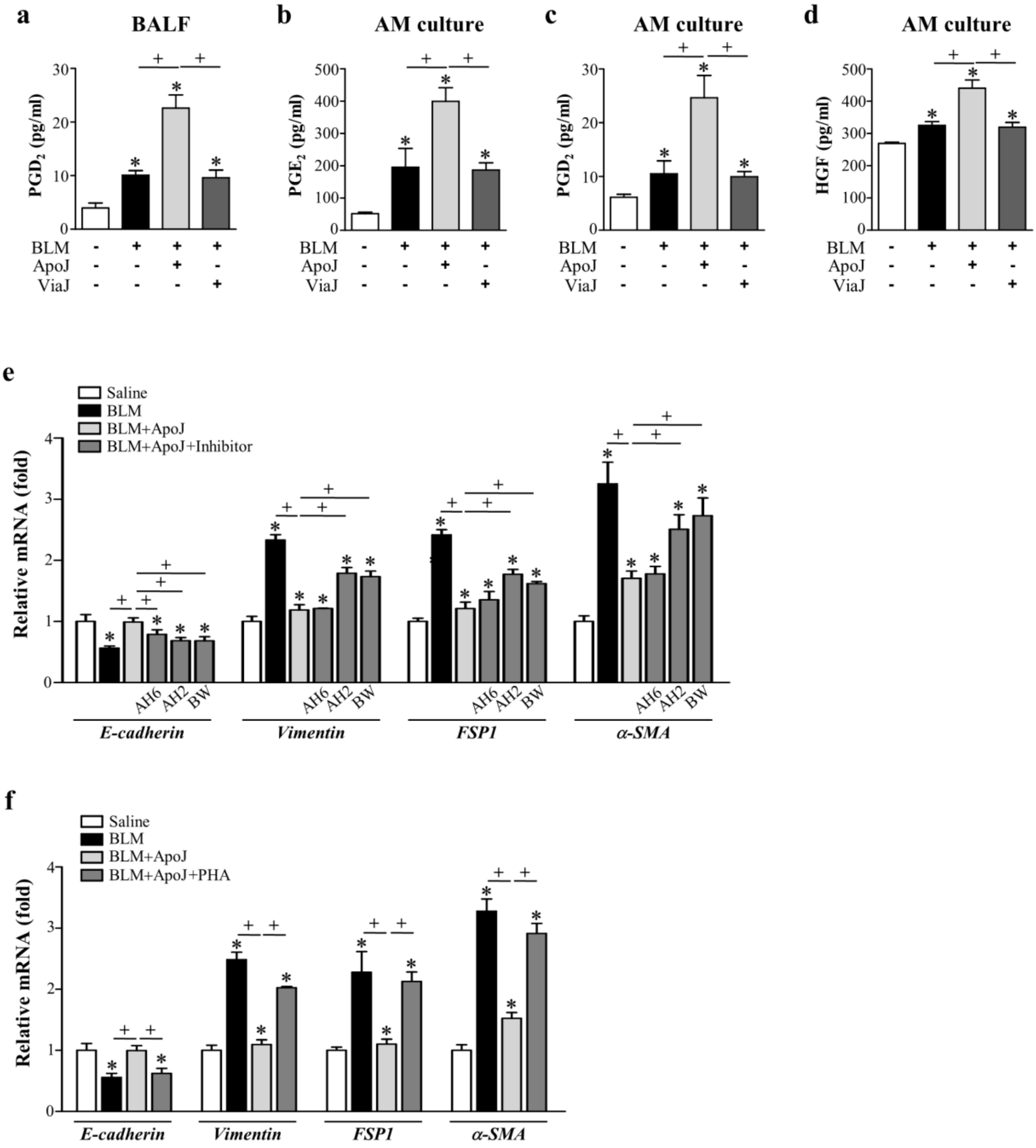
a
BALF
PGD2 (pg/ml)
b
AM culture
PGE2 (pg/ml)
c
AM culture
PGD2 (pg/ml)
d
AM culture
HGF (pg/ml)
BLM
ApoJ
ViaJ
e
Saline
BLM
BLM+ApoJ
BLM+ApoJ+Inhibitor
Relative mRNA (fold)
AH6
AH2
BW
E-cadherin
Vimentin
FSP1
α-SMA
f
Saline
BLM
BLM+ApoJ
BLM+ApoJ+PHA
Relative mRNA (fold)
E-cadherin
Vimentin
FSP1
α-SMA

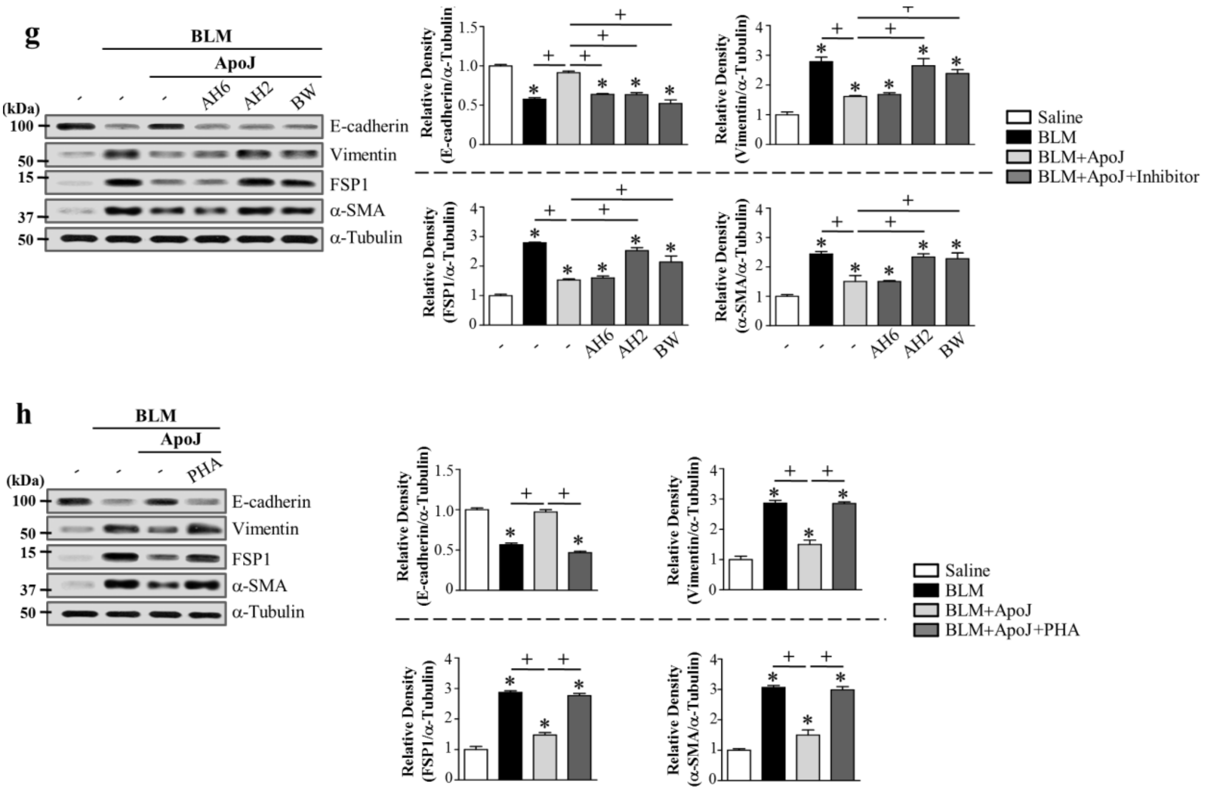

Figure 10. Reduction of EMT phenotype by *in vivo* instillation of apoptotic cells. (a-d) Two days after bleomycin (BLM) treatment, lungs were instilled with saline alone (Sal), viable Jurkat cells (ViaJ), or apoptotic Jurkat cells (ApoJ) intratracheally. Mice were euthanized at 14 days following BLM treatment. PGD_2 levels in the BAL fluid (a), PGE_2 (b), PGD_2 (c), and HGF levels (d) in alveolar macrophages were quantified by ELISA. (e) Where indicated, 5 mg/kg AH-6809 (AH6, i.p.), 10 mg/kg AH-23848 (AH2, i.p.), or 1 mg/kg BW-A868C (BW, s.c.) was administered simultaneously with ApoJ instillation 2 days after BLM treatment and every day thereafter. Mice were euthanized at 14 days following BLM treatment. (f) PHA-665752 (PHA, 25 mg/kg) was administered intraperitoneally at the same time with ApoJ instillation 2 days after BLM treatment and every 2 days for days 10 to 20 after BLM treatment. Mice were euthanized at 21 days following BLM treatment. (e, f) The amounts of *E-cadherin*, *vimentin*, *FSP1*, or *α-SMA* mRNAs in lung samples were analyzed by real-time PCR and normalized to that of *Hprt* mRNA. (g, h) Immunoblots of total cell lysates were performed with anti-E-cadherin, vimentin, FSP1, or α-SMA antibodies. Densitometric analysis of the indicated EMT markers' relative abundances. Values represent the mean ± s.e.m. from five mice in each group. $^*P < 0.05$ compared with control; $^+P < 0.05$ as indicated.
